# Distinct gut microbiomes in two polar bear subpopulations inhabiting different sea ice ecoregions

**DOI:** 10.1038/s41598-021-04340-2

**Published:** 2022-01-11

**Authors:** Megan Franz, Lyle Whyte, Todd C. Atwood, Kristin L. Laidre, Denis Roy, Sophie E. Watson, Esteban Góngora, Melissa A. McKinney

**Affiliations:** 1 Department of Natural Resource Sciences, McGill University, Sainte-Anne-de-Bellevue, QC H9X 3V9 Canada; 2 United States Geological Survey (USGS), Alaska Science Center, University Drive, Anchorage, AK 99508 USA; 3 Polar Science Center, Applied Physics Laboratory, University of Washington, Seattle, WA USA; 4 Greenland Institute of Natural Resources, P.O. Box 570, Nuuk, Greenland; 5 School of Biosciences, Cardiff University, The Sir Martin Evans Building, Museum Avenue, Cardiff, UK

**Keywords:** Ecology, Microbiology

## Abstract

Gut microbiomes were analyzed by 16S rRNA gene metabarcoding for polar bears (*Ursus maritimus*) from the southern Beaufort Sea (SB), where sea ice loss has led to increased use of land-based food resources by bears, and from East Greenland (EG), where persistent sea ice has allowed hunting of ice-associated prey nearly year-round. SB polar bears showed a higher number of total (940 vs. 742) and unique (387 vs. 189) amplicon sequence variants and higher inter-individual variation compared to EG polar bears. Gut microbiome composition differed significantly between the two subpopulations and among sex/age classes, likely driven by diet variation and ontogenetic shifts in the gut microbiome. Dietary tracer analysis using fatty acid signatures for SB polar bears showed that diet explained more intrapopulation variation in gut microbiome composition and diversity than other tested variables, i.e., sex/age class, body condition, and capture year. Substantial differences in the SB gut microbiome relative to EG polar bears, and associations between SB gut microbiome and diet, suggest that the shifting foraging habits of SB polar bears tied to sea ice loss may be altering their gut microbiome, with potential consequences for nutrition and physiology.

## Introduction

Many metabolic and immune system processes of higher-order organisms are carried out by an assemblage of microbes—predominantly bacteria—found within their gastrointestinal systems^1,2^. Thus, the gut microbiome influences host nutrition, health, and resistance to enteric pathogenic diseases^1,3,4^. Although far less studied than those of human, laboratory, or domestic animals^5^, the gut microbiomes of many wild animal species have recently been characterized^6^. Many of these species host Firmicutes, Bacteroidetes, Proteobacteria, Actinobacteria and Verrucomicrobia as the major bacterial phyla^1^. Yet, differences in the species composition within phyla among host species appears to be the norm^7^. In mammalian wildlife, variation in bacterial community composition among host species has been attributed to a combination of host phylogeny, habitat, and diet^2,8,9^; however, diet appears to be a predominant driver of interspecific variation in gut bacterial community composition and of intraspecific variation^10–12^.

Recent research has argued that inter-individual variation can provide insight into the adaptive potential of wildlife species faced with environmental stressors^13,14^. Yet, the role of multiple drivers (i.e., host sex, age, diet, and body condition) on inter-individual and inter-population variation among wild animal hosts in terms of their gut bacterial communities is understudied^8,9,15,16^. In a small study on wild bears, for example, four individual grizzly bears (*Ursus arctos*) from a population in Alberta, Canada, feeding in part on agricultural subsidies (cereals, domestic animals) showed significant differences in genus-level bacterial abundance compared to four grizzly bears from a population hunting wild prey (e.g., ungulates); both populations also showed wide variation among individuals and differences in gut bacteria compared to two captive grizzly bears^17^. In 16 individuals of *U. arctos* from Europe, changes in individual gut bacterial diversity and composition occurred among individuals between hibernation and active periods^18^. Thus, differences in the gut microbial community both within and among populations can shed light into the consequences of changes in habitat use—such as exposure to different environmental microbes, macrofauna, and climate factors that can influence microbial presence/abundance in a region—as well as differences in feeding habits within wild species.

**Figure 1:**
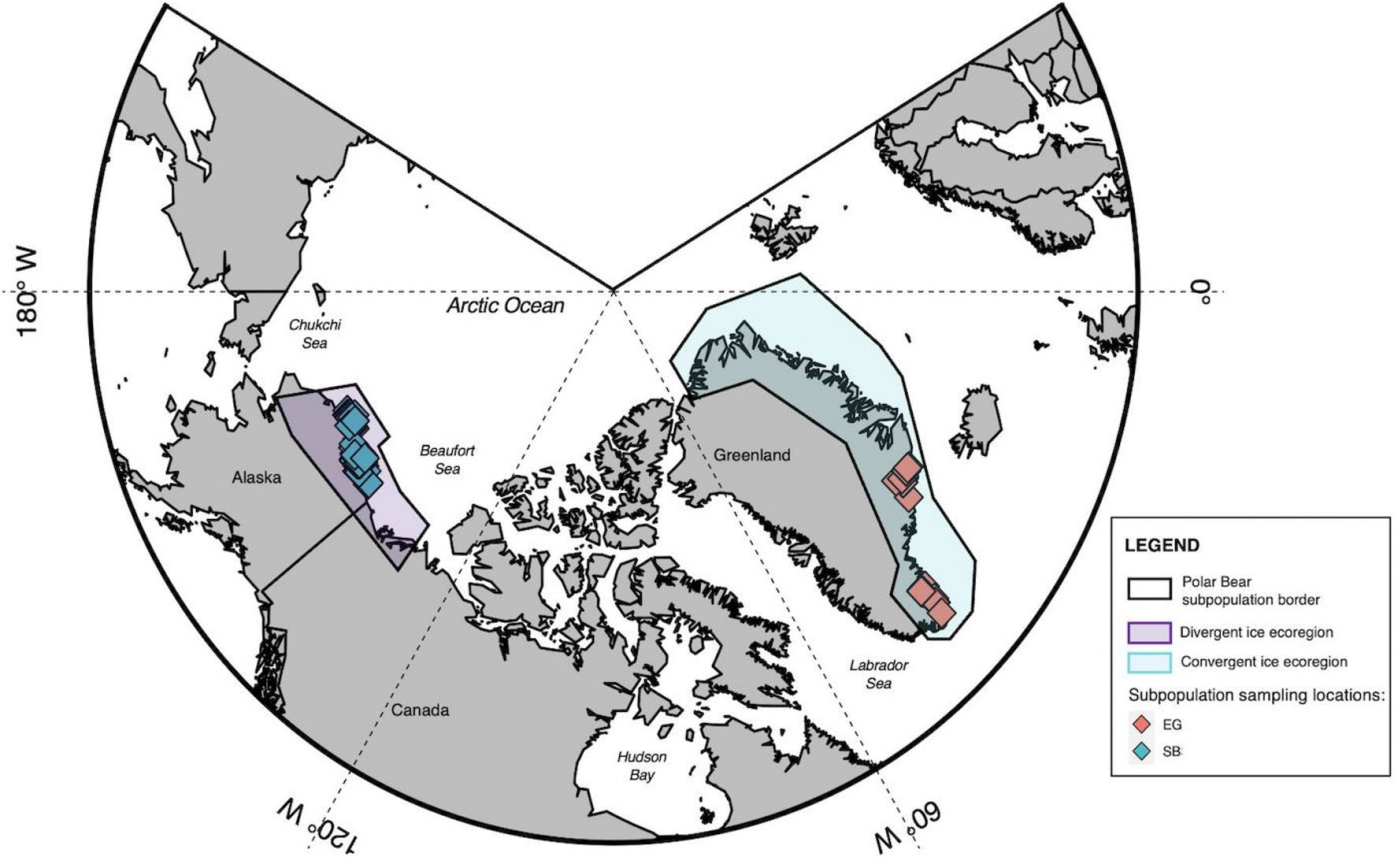
Map of sampling locations for the two polar bear subpopulations in this study. The East Greenland (EG) subpopulation is distributed along the east Greenland shoreline and occurs in a convergent ice ecoregion (blue), while the Southern Beaufort Sea (SB) polar bear subpopulation is distributed along the northern shore of Alaska and Canada and occurs in a divergent ice ecoregion (purple). Map made with R Studio using the PlotSvalbard package in R (V. 4.0.3).

Polar bears (*Ursus maritimus*) are distributed across the circumpolar Arctic in nineteen spatially segregated subpopulations^19^. Similarity in habitats among some of these subpopulations has allowed for their classification into ecoregions, each of which has distinct sea ice characteristics that influence polar bear seasonal movements, foraging activities, and diets^20–22^. The ‘convergent ecoregion’ tends to receive supplemental sea ice formed within other regions and the Arctic Basin, providing polar bears, such as the East Greenland (EG) subpopulation, with near year-round access to sea ice and to the ice seals that comprise most of their diet^20,23^. Within the divergent ecoregion, including polar bears in the Southern Beaufort Sea (SB) subpopulation, sea ice was present year-round before the 1980s^24^ (Fig. 1). However, with climate change-mediated loss of sea ice over the last four decades, SB polar bears now spend longer periods of time onshore during the reduced ice season^25,26^. This has led to increased access to onshore foods, including blubber, meat, and bones of bowhead whales leftover from local subsistence harvests (‘bone piles’), as well as carcasses of fish, caribou, and birds left nearby^27^.

The distinct sea ice conditions that result in differing habitat use and feeding habits for EG and SB polar bears provides a unique opportunity to explore inter-population variation in the gut microbiota of a wild animal species and could provide insight into the ability of polar bears to cope with added environmental stressors introduced by climate change. Preliminary findings on the gut microbiota of a single polar bear subpopulation using 16S rRNA gene clone libraries detected just one phylum, *Firmicutes*, suggesting low gut bacterial diversity relative to other mammalian species^28^. However, more recently, 16S rRNA metataxonomics using Illumina technology approaches found 25 bacterial phyla in the SB subpopulation and greater gut bacterial diversity for bears that spend part of the year onshore and that likely have a more diverse terrestrial-based diet relative to bears remaining offshore with likely narrower diets consisting largely of ice seals^29^. In this study, we use high-throughput 16S rRNA gene amplicon sequencing techniques to assess inter-population variation in gut microbial composition and diversity between EG and SB polar bears using samples collected during the same season (late-winter/early spring). We also explore how sex/age class, body mass (as an indicator of body condition), and (for SB bears) dietary patterns based on fatty acid (FA) signatures^30^, are associated with inter-and (for SB bears) intra-population variation in gut microbial communities in two wild polar bear subpopulations. Given the evidence of dietary alterations occurring in the SB subpopulation and the distinct ice ecoregion differences that force some SB polar bears to spend greater amounts of time on land, we predict that SB gut microbiota will be more diverse and that we will see a higher degree of interindividual variation and a greater number of overall and unique bacterial species in the SB subpopulation compared to EG. We also expect that diet will be a significant driver of both gut bacterial diversity and composition in the subset of SB polar bears for which FA data was available.

## Results

### Gut bacterial diversity and composition of EG and SB polar bears

A total of 12,294,006 reads were obtained for both EG (*n* = 34) and SB (*n* = 59) samples combined, with an average of 81,960 reads per sample. Following DADA2 processing, 6,172 amplicon sequence variants (ASVs) were identified overall, which were then further reduced to 1129 ASVs after removing ASVs with less than two counts and zero variance across all samples.

Although mean alpha diversity was qualitatively higher in SB than in EG polar bears for Shannon (SB: 2.74 +/− 0.06; EG: 2.65 +/− 0.07), Inverse Simpson (SB: 9.2 +/− 0.6; EG: 8.3 +/− 0.6), and Faith’s Phylogenetic Diversity (SB: 13.3 +/− 0.4; EG: 12.9 +/− 0.5) (Supplementary Fig. S1), linear models showed no effect of subpopulation for any of these alpha diversity indices (Supplementary Table S1).

**Figure 2:**
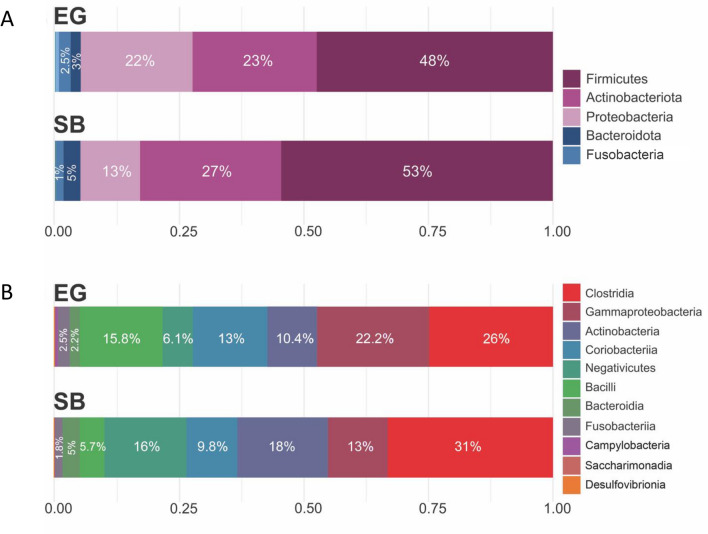
(**A**) Relative abundance bar plot showing the five most abundant bacterial phyla, averaged across all samples within each subpopulation (East Greenland [EG] and Southern Beaufort Sea [SB]) and (**B**) Relative abundance bar plot showing the eight most abundant bacterial classes, averaged across all samples within each subpopulation.

**Figure 3:**
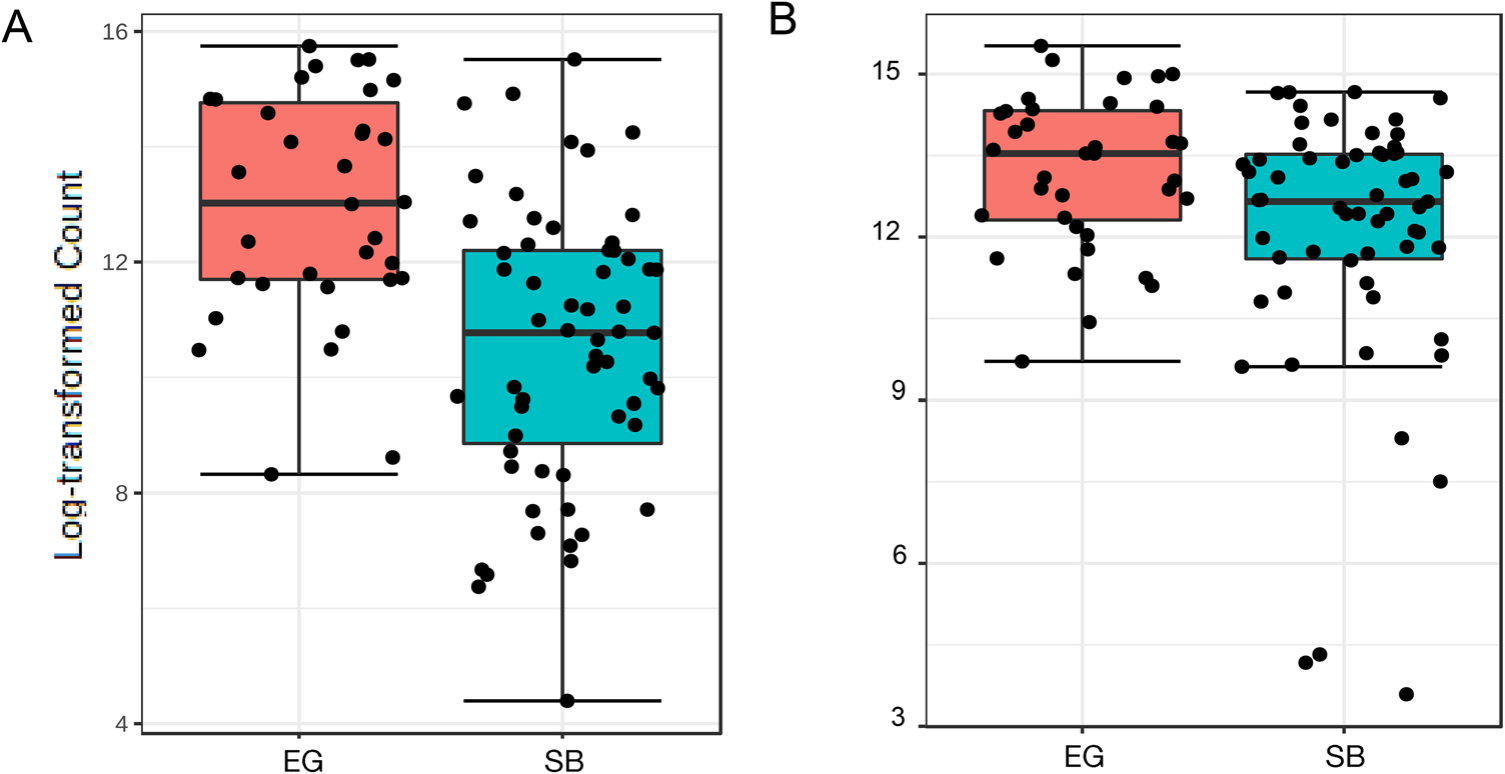
Boxplots of log-transformed counts for bacterial classes showing differential abundances of (**A**) Bacilli, significantly higher in East Greenland (EG) than Southern Beaufort Sea (SB) polar bears (Group means: EG: 13.0 ± 0.3; SB: 10.5 ± 0.3) (**B**) Coriobacteria, significantly higher in EG than in SB polar bears (Group means: EG: 13.1 ± 0.2; SB: 12.0 ± 0.3). Analysis of composition with bias correction (ANCOM-BC) test results summarized in Supplementary Table S2.

Differences in composition between EG and SB polar bears were assessed at multiple bacterial taxonomic levels—Phylum, Class, Genus, and ASV—and found to differ significantly at bacterial class (*R*^2^ = 0.035, *F*_*1,93*_ = 3.43, *p* = 0.008), genus (*R*^2^ = 0.046, *F*_*1,93*_ = 4.62, *p* < 0.001), and ASV-levels (*R*^2^ = 0.052, *F*_*1,93*_ = 5.20, *p* < 0.001) (Table 1). Of the seventeen detected phyla, five were predominant comprising ~ 97% of the total reads (Fig. 2A). Of the 24 classes detected, eight accounted for 99% of total reads in both polar bear subpopulations and varied in their proportional contributions among the two subpopulations (Fig. 2B). *Post-hoc* analysis of composition with bias correction (ANCOMBC) testing found that the abundances of two bacterial classes, Bacilli and Coriobacteria, differed significantly between EG and SB polar bears (Fig. 3, Supplementary Table S2).

**Table 1:** Summary of permutational analysis of variance (PERMANOVA) results* assessing differences in gut bacterial composition at bacterial phylum, class, genus, and ASV levels for East Greenland (EG) and Southern Beaufort Sea (SB) polar bear subpopulations using Bray–Curtis distance method.

Phylum-level	Analysis of Variance Table					
	*Df*	*SumsOfSqs*	*MeanSqs*	*F.Model*	*R2*	*Pr(*> *F)*
Sex/age class	3	0.270	0.090	1.370	0.044	0.210
Subpopulation	1	0.141	0.141	2.143	0.023	0.103
Body Condition	1	0.053	0.053	0.802	0.008	0.485
Subpopulation: Body Condition	1	0.087	0.087	1.320	0.014	0.268
Residuals	86	5.653	0.066	NA	0.911	NA
Total	92	6.203	NA	NA	1.000	NA

Class-level	Analysis of Variance Table					
	*Df*	*SumsOfSqs*	*MeanSqs*	*F.Model*	*R2*	*Pr(*> *F)*
**Sex/age class**	**3**	**1.058**	**0.353**	**2.511**	**0.076**	**0.004**
**Subpopulation**	**1**	**0.481**	**0.481**	**3.429**	**0.035**	**0.008**
Body Condition	1	0.089	0.089	0.635	0.006	0.665
Subpopulation: Body Condition	1	0.240	0.240	1.712	0.017	0.136
Residuals	86	12.073	0.140	NA	0.866	NA
Total	92	13.942	NA	NA	1.000	NA

Genus-level	Analysis of Variance Table					
	*Df*	*SumsOfSqs*	*MeanSqs*	*F.Model*	*R2*	*Pr(*> *F)*
**Sex/age class**	**3**	**2.077**	**0.692**	**2.658**	**0.079**	** < 0.001**
**Subpopulation**	**1**	**1.212**	**1.212**	**4.654**	**0.046**	** < 0.001**
Body Condition	1	0.151	0.151	0.579	0.006	0.881
Subpopulation: Body Condition	1	0.328	0.328	1.261	0.013	0.219
Residuals	86	22.400	0.260	NA	0.856	NA
Total	92	26.168	NA	NA	1.000	NA

ASV-level	Analysis of Variance Table					
	*Df*	*SumsOfSqs*	*MeanSqs*	*F.Model*	*R2*	*Pr(*> *F)*
**Sex/age class**	**3**	**2.172**	**0.724**	**2.731**	**0.081**	** < 0.001**
**Subpopulation**	**1**	**1.391**	**1.391**	**5.246**	**0.052**	** < 0.001**
Body Condition	1	0.157	0.157	0.592	0.006	0.852
Subpopulation: Body Condition	1	0.299	0.299	1.129	0.011	0.321
Residuals	86	22.798	0.265	NA	0.850	NA
Total	92	26.817	NA	NA	1.000	NA

*Significant terms are in bold.

**Figure 4:**
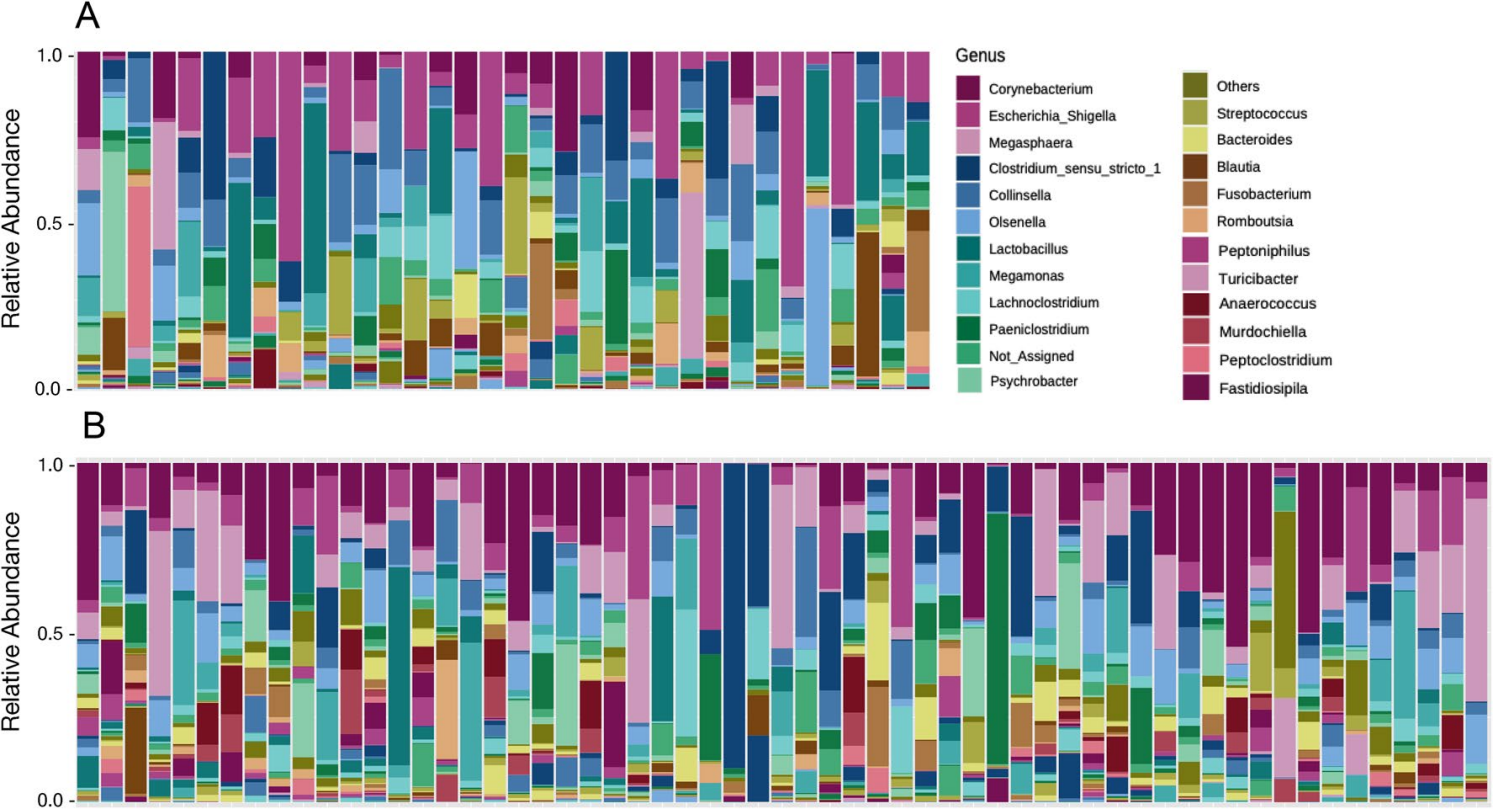
Relative abundance bar plots at genus level showing extent of interindividual variation among polar bears in the (**A**) East Greenland (EG) and (**B**) Southern Beaufort Sea (SB) subpopulations.

**Figure 5:**
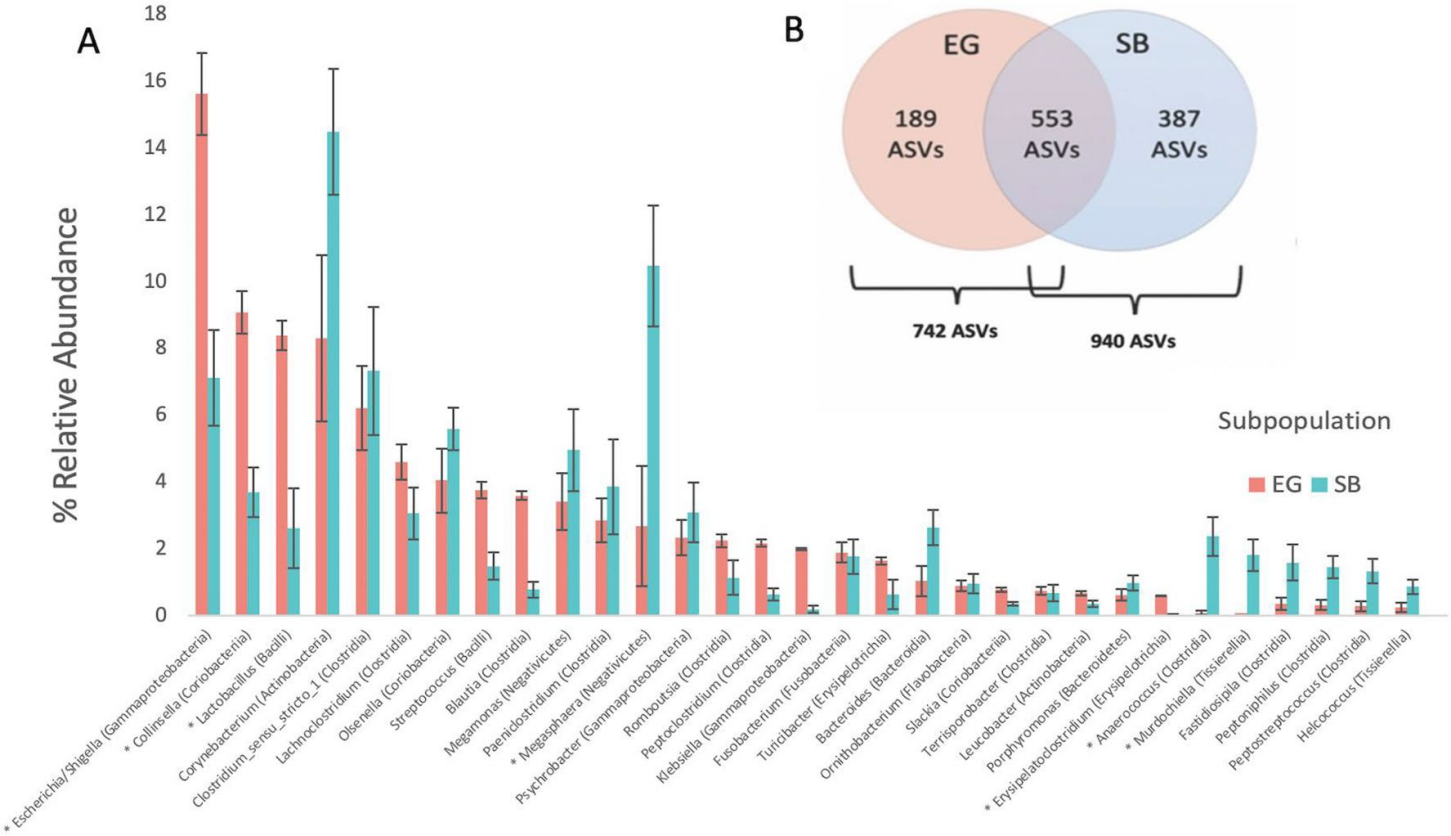
(**A**) Grouped bar plot showing the top 31 most abundant bacterial genera detected in East Greenland (EG) and Southern Beaufort Sea (SB) polar bear subpopulations (associated bacterial class noted in parentheses). Relative abundances were averaged across all samples within each subpopulation (EG and SB). Asterisks (*) indicate genera with significantly different abundances between the two subpopulations (see Supplementary Table S3 for statistical results obtained using analysis of composition with bias correction (ANCOM-BC) approach). (**B**) Venn diagram insert showing number of shared, unique, and total amplicon sequence variants (ASVs) detected in EG and SB polar bear subpopulations.

Of 203 total genera detected, 31 (the combined top 25 genera from each subpopulation) comprised ~ 90% of all reads for EG and SB bears and 12 were unique to EG while 51 were unique to SB. Despite observable inter-individual variation at the genuslevel for both EG and SB polar bears (Fig. 4, Table 1) *post-hoc* ANCOMBC analysis found that the abundances of seven of the top 31 most abundant genera still differed significantly between EG and SB bears (Fig. 5A, Supplementary Table S3). The remaining 13 differentially abundant genera are listed in Supplementary Table S3.

**Figure 6:**
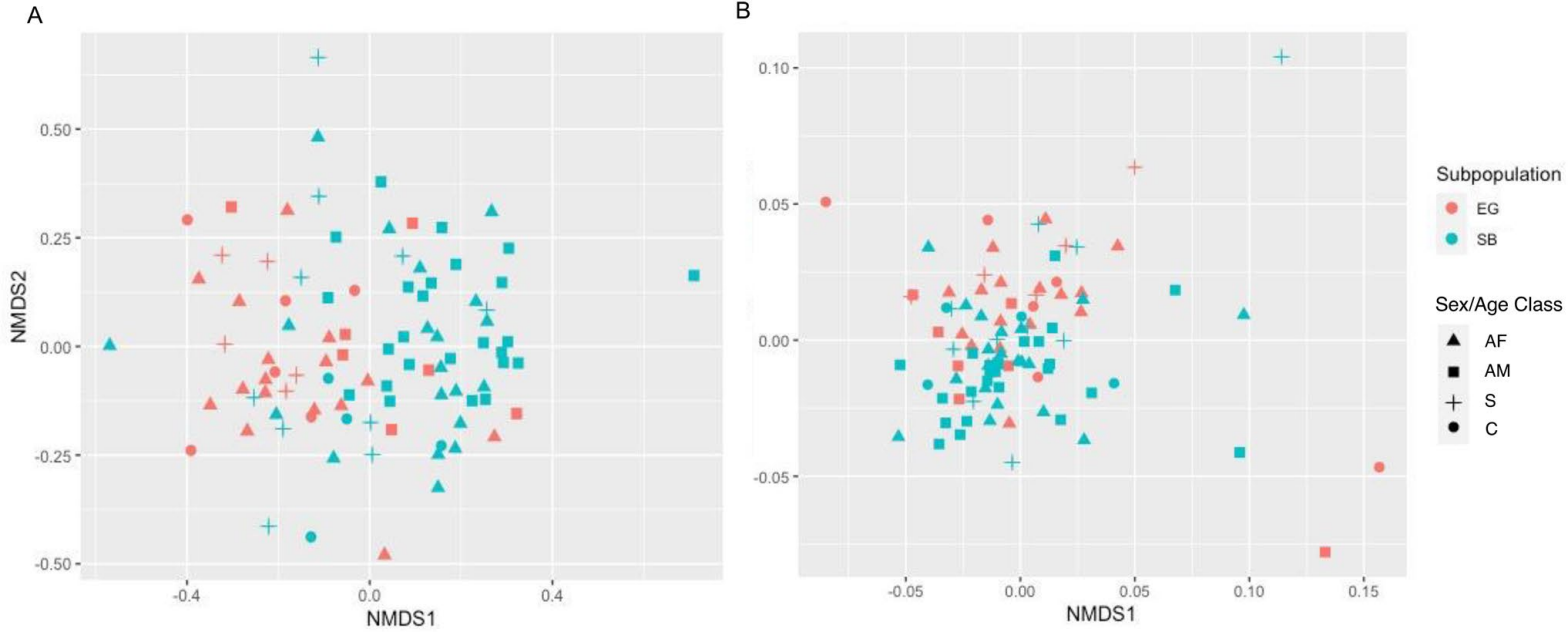
Non-metric multi-dimensional scaling (NMDS) plots showing gut bacterial communities for East Greenland (EG) and Southern Beaufort Sea (SB) polar bear subpopulations with color denoting subpopulation affiliation and shapes denoting Sex/Age Classes (adult females [AF], adult males [AM] and subadults [S] compared to cubs [C]) determined using (**A**) Bray–Curtis (Stress = 0.26; PERMANOVA: R^2^ = 0.06, *p* = 0.001; PERMDISP: *p* = 0.5) and (**B**) weighted UniFrac distances (Stress = 0.16; PERMANOVA: R^2^ = 0.06, *p* = 0.001; PERMDISP: *p* = 0.5) calculated at amplicon sequence variant (ASV)-level. Points represent individual fecal samples.

A total of 742 ASVs were detected in EG polar bears and 940 ASVs were found for SB polar bears (Fig. 5B). Of the 553 shared ASVs, 48 differed significantly in their abundances between subpopulations (Supplementary Table S4). Significant differences in composition at the ASV level were found between subpopulations using both Bray–Curtis distances (PERMANOVA: *R*^*2*^ = 0.052, *F*_*1,93*_ = 5.20, *p* < 0.001) (Fig. 6A) and the phylogenetic Weighted UniFrac Distances (PERMANOVA: *R*^*2*^ = 0.065, *F*_*1,93*_ = 6.49, *p* = 0.001) (Fig. 6B). These results were not confounded by heterogeneity of subpopulation group dispersions (Bray–Curtis: PERMDISP: *F*_*1,93*_ = 0.75, *p* = 0.39; Weighted UniFrac: PERMDISP: *F*_*1,93*_ = 0.61, *p* = 0.44).

### Influence of sex/age class and body condition on gut bacterial diversity and composition in EG and SB polar bears

Neither sex/age class, body condition, nor any interaction terms significantly explained variation in Shannon and Inverse Simpson alpha diversity, although sex/age class was found to be a near-significant term in the linear model explaining variation in Faith’s Phylogenetic Diversity (Supplementary Table S1, Supplementary Fig. S2).

Significant differences in gut bacterial composition were found between polar bears of different sex/age classes (i.e. adult females [AF], adult males [AM], subadults [S], and cubs [C]) at the class (PERMANOVA: *R*^*2*^ = 0.076, *F*_*1,93*_ = 2.51, *p* = 0.004), genus (*R*^*2*^ = 0.079, *F*_*1,93*_ = 2.66, *p* < 0.001) and ASV-levels (Bray–Curtis distance: *R*^*2*^ = 0.081, *F*_*1,93*_ = 2.73, *p* < 0.001; Weighted UniFrac distance: *R*^*2*^ = 0.079, *F*_*1,93*_ = 2.53, *p* = 0.002) (Table 1, Supplementary Fig. S3). However, the assumption of homogeneity of multivariate group dispersions for the sex/age class groups was not met for either the Bray–Curtis or Weighted UniFrac indices at ASV-level (PERMDISP: *p* = 0.005 and *p* = 0.041, respectively), so results should be interpreted with some caution. *Post-hoc* ANCOMBC results showed that the abundances of three bacterial classes (Bacilli, Parcubacteria, and Saccharimonadia) (Supplementary Fig. S4), 21 bacterial genera and 65 ASVs differed significantly among the different sex/age class groups (Supplementary Tables S5, S6 and S7). There were no significant effects of body condition or any of the interaction terms at any taxonomic level (Table 1).

### Influence of diet as a driver of gut bacterial diversity and composition in SB polar bears

For a subset of SB polar bears (*n* = 46), diet data was obtained using fatty acid (FA) signature analysis. The proportions of key dietary FAs were used in a principal components analysis (PCA) to reduce the number of variables from the fatty acids to just two principal components, which explained 83.3% of the total variation in polar bear FA signatures. Diet was represented in subsequent microbiome models by the individual’s scores along FA_PC1 and FA_PC2 (Supplementary Fig. S5). PERMANOVAs and multiple linear regression models were run to assess how diet influences gut bacterial diversity and composition, respectively. FA_PC1 was a significant term in models explaining variation in Shannon and Inverse Simpson indices of alpha diversity for these bears and FA_PC2 was also a nearly-significant term in the model explaining differences in composition (Table 2, Supplementary Table S8). Athough diet did not explain variation in gut bacterial composition at class-level, significant effects of diet (FA_PC1 and FA_PC2) were found at bacterial genus-level and ASV-levels (Table 3).

**Table 2:** Summary of top models showing influence of diet (FA_PC1 and FA_PC2 axes) and other relevant metadata on variation in alpha diversity indices (Shannon, Inverse Simpson, Faith’s phylogenetic distance) and beta diversity indices (Bray–Curtis and weighted UniFrac distances) for the subset of Southern Beaufort Sea (SB) polar bears for which diet data was available. There were no significant terms in models explaining variation using weighted UniFrac beta diversity axes.

Diversity index	Top Model	F	*P*	Mult R^2^	Adj. R^2^
Shannon	~ Sex/age class* + Body Condition + FA_PC1* + FA_PC2	2.73	0.033	0.25	0.16
Inverse Simpson	~ FA_PC1* + FA_PC2	3.51	0.039	0.14	0.10
Faiths Phylogenetic Diversity	~ Sex/age class* + FA_PC1	5.48	0.003	0.28	0.23
Bray Curtis (NMDS1)	~ Sex/age class *	5.58	0.007	0.21	0.17
Bray Curtis (NMDS2)	~ FA_PC2 . + Capture year	3.16	0.035	0.18	0.13
Weighted UniFrac (NMDS1)	~ 1 (NULL)	–	–	–	–
Weighted UniFrac (NMDS2)	~ 1 (NULL)	–	–	–	–

**Table 3:** Summary of permutational analysis of variance (PERMANOVA) results* assessing differences in gut bacterial composition at bacterial class, genus, and amplicon sequence variant (ASV)-levels for the subset of Southern Beaufort Sea (SB) polar bears using Bray–Curtis distances.

Class-level	Analysis of Variance Table					
	*Df*	*SumsOfSqs*	*MeanSqs*	*F.Model*	*R2*	*Pr(*> *F)*
Sex/age class	2	0.319	0.160	1.274	0.053	0.233
Body Condition	1	0.209	0.209	1.670	0.035	0.142
FA_PC1	1	0.126	0.126	1.005	0.021	0.411
**FA_PC2**	**1**	**0.262**	**0.262**	**2.092**	**0.043**	**0.085**
**Capture year**	**1**	**0.261**	**0.261**	**2.080**	**0.043**	**0.074**
Residuals	39	4.886	0.125		0.806	
Total	45	6.063			1	

Genus-level	Analysis of Variance Table					
	*Df*	*SumsOfSqs*	*MeanSqs*	*F.Model*	*R2*	*Pr(*> *F)*
**Sex/age class**	**2**	**0.810**	**0.405**	**1.776**	**0.071**	**0.023**
Body Condition	1	0.209	0.208	0.914	0.018	0.527
**FA_PC1**	**1**	**0.504**	**0.504**	**2.208**	**0.044**	**0.024**
**FA_PC2**	**1**	**0.439**	**0.439**	**1.925**	**0.039**	**0.034**
**Capture year**	**1**	**0.483**	**0.483**	**2.118**	**0.043**	**0.019**
Residuals	39	8.895	0.228		0.784	
Total	45	11.339			1	

ASV-level	Analysis of Variance Table					
	*Df*	*SumsOfSqs*	*MeanSqs*	*F.Model*	*R2*	*Pr(*> *F)*
**Sex/age class**	**2**	**0.719**	**0.359**	**1.568**	**0.063**	**0.057**
Body Condition	1	0.205	0.205	0.894	0.018	0.519
**FA_PC1**	**1**	**0.557**	**0.557**	**2.431**	**0.049**	**0.009**
**FA_PC2**	**1**	**0.442**	**0.442**	**1.927**	**0.039**	**0.023**
**Capture year**	**1**	**0.485**	**0.485**	**2.116**	**0.043**	**0.024**
Residuals	39	8.937	0.229		0.788	
Total	45	11.344			1	

*Significant terms are in bold**.**

Similar to the analyses including both EG and SB subpopulations, sex/age class significantly explained variation in gut bacterial diversity and composition among SB polar bears (Tables 2, 3, see Supplementary Text 1). Additionally, body condition was found to be a nearly-significant term in the Shannon alpha diversity model and capture year was found to be a significant term in the model for the Bray–Curtis NMDS2 axis (Table 2, Supplementary Table S8). Capture year was also found to be a significant term in composition PERMANOVAs using Bray–Curtis distance method (Table 3). There were no significant terms in models explaining variation in the Weighted UniFrac NMDS axes (Table 2, Supplementary Table S8).

## Discussion

Polar bears from the SB subpopulation showed significant differences in gut bacterial composition at multiple bacterial taxonomic levels compared to EG polar bears and an overall greater number of unique and total bacterial genera and ASVs. The particular bacterial classes and genera which were elevated in one subpopulation versus the other were consistent with a potentially altered and more varied gut microbiota in the more land-associated SB subpopulation relative to the more sea ice-based EG subpopulation. Relative to SB polar bears, those in the EG subpopulation had higher levels of bacteria from the class Bacilli, which has been suggested to play an important role in restoring gut health and maintaining gut homeostasis^31^ and from the class Coriobacteria which is a typical taxonomic group found in the human gut and known to play a role in gut microbiome health^32^. Although many of the most abundant bacterial genera were shared between the two subpopulations, some genera were significantly higher in EG bears compared to SB bears. Specifically, *Collinsella*, *Lactobacillus, Erysipelatoclostridium, and Escherichia-Shigella* were higher in EG, and some of these genera have important probiotic properties, at least based on human studies^33–36^. Bacteria from class Coriobacteria (e.g. *Collinsella*) have been suggested to aid with lipid metabolism in human studies^37^ and with cholesterol metabolism in controlled studies on hamsters^38^. These differences could imply that EG bears have a healthier ‘baseline’ gut microbiome compared to SB bears, a reflection of their likely narrower dietary niche breadth and continued access to traditional lipid-rich prey species, however this is difficult to conclude given the lack of studies on functional roles of these bacteria in wildlife^5^. Alternatively, the differential bacterial classes and genera between SB and EG bears could simply reflect local regional adaptations based on differences in food availability and other geographic and ecosystem variables such as exposure to sea ice vs. terrestrial habitat, exposure to different macro- and micro- fauna, etc.

Some bacterial classes and genera were elevated in SB polar bears compared to EG polar bears. Two genera *Megasphaera* and *Megamonas*, which contributed to ~ 15% of class Negativicutes reads within the SB subpopulation, were elevated in SB compared to EG polar bears (*Megasphaera* was significantly elevated) and may be important components of rumen microbiomes. Further, some Negativicutes species have metabolic properties related to the breakdown of polysaccharides and lactate into short chain fatty acids (SCFAs) which have been suggested to promote gut health^39–42^. This observation of elevated Negativicutes could potentially indicate increases in carbohydrates or starches in SB diets related to inputs from terrestrial foods, such as berries, which polar bears have been observed to eat while onshore^43,44^. Bacteroidia were also elevated in SB relative to EG polar bears. Two genera of Bacteroidia comprised ~ 4% of reads for SB bears (compared to ~ 1.6% in EG bears): *Bacteroidetes* and *Porphyromonas*. *Bacteroidetes* have been described in human microbiome studies as having complex metabolic roles covering plant and polysaccharide degradation, protein metabolism, or just as a component of healthy adult gut microbiota^45^. Changes in abundance (i.e., increases or decreases) of *Bacteroidetes* have also been associated with several GI tract diseases, such as obesity and irritable bowel syndrome in humans^46–49^. *Porphyromonas* species are asaccharolytic and are often associated with the oral microbiome and can occasionally become pathogenic^50–52^. Finally, a few genera that were significantly higher in SB compared to EG bears, (e.g., *Megasphaera*, *Anaerococcus*) are typically part of the commensal microbiota. However, *Anaerococcus* has been linked to polymicrobial infections and can become pathogenic in humans or human-associated microbiomes^53–56^. While some of these bacterial genera that are more abundant in SB bears compared to EG bears have been previously linked to adverse health effects in human and controlled studies, they could also simply reflect a more varied and diverse diet for SB polar bears which would necessitate a shift in metabolic function of the gut microbiome.

In general, the characteristics and functions of specific bacteria can vary depending on host species. As such, these bacteria might serve different functional roles in the polar bear gut microbiome compared to what has been shown in studies on the gut microbiomes of humans and other mammalian species. Further, higher or lower gut bacterial diversity and the presence or introduction of novel bacterial species could ultimately lead to the development of an adaptive gut microbiome, particularly when considering potential shifts toward protein and carbohydrate metabolism type functions of the bacterial species that are increased in the SB subpopulation. Alternatively, it could lead to gut dysbiosis and negative health consequences for an individual, population, or species^57^. While it is difficult to predict any long-term consequences that could result from these observed differences in bacterial composition and diversity between the SB and EG subpopulations, it will be important to continue to monitor such changes and investigate their health consequences.

Additional factors could be contributing to these compositional differences between subpopulations, such as host phylogeny, immune system effects, and environmental differences (biogeography, variety of cohabitating species present in the region, etc.)^1,4,58–60^. Although our dataset did not contain a sufficient number of capture years for both subpopulations to evaluate climate and ecological variation that could influence temporal trends in the gut microbiota, future work with additional years of collection data should assess this relationship. Nonetheless, differences in diet are likely important in explaining much of the differences in the gut microbiome between EG and SB bears, given the importance of diet in driving gut microbiome composition and separate studies pointing to dietary differences between these subpopulations^2,8–10,17^. In response to climate change, SB bears show increased use of terrestrial habitat and terrestrially-based food resources in the late summer and fall months^61–63^. Reduced access to ice seal prey has been tied to declines in the SB polar bear population^64^ and other studies speculate that alternative food resources will likely be nutritionally insufficient for polar bears^26,63^ which could have serious implications for long-term persistence of the species. Any changes in the gut microbiome could potentially impact immune functioning or impair nutrient uptake for polar bears in this region, further exacerbating these existing stressors faced by the SB subpopulation in a period of continued sea ice decline and habitat loss. Consumption of non-traditional prey species and tissue types also likely exposes them to novel pathogens (Watson et al., submitted) and gut microbiota, either via contact with other scavenging species or through changes in macronutrients of their prey^63,65^.

This hypothesis is supported by findings of higher gut bacterial diversity in onshore versus offshore polar bears in the SB subpopulation^29^. Although we did not have seasonal metadata distinguishing onshore vs. offshore SB bears as all fecal samples were collected in the spring before sea ice breakup, these differences in inter-individual foraging behavior likely contribute to increased rare/unique ASVs detected and overall larger variance in beta diversity within the SB subpopulation compared to the EG subpopulation. FA signatures from adipose samples collected in winter-spring of 1984–2011 suggests that EG polar bears mainly consume ice seals, and probably largely in the form of seal blubber. This is despite the proportion of Arctic seals such as ringed seals (*Pusa hispida)* declining, while the proportion of northward range-shifting sub-Arctic seals such as harp seals (*Phoca groenlandica)* and hooded seals (*Crystophora cristata*), increased^30^. Thus, while there is evidence that EG polar bears show decreased consumption of traditional ringed seal prey, the overall change in macronutrient composition between these prey types may be low (i.e., still predominantly blubber lipids) and could partially explain the detection of fewer bacterial genera and ASVs in EG compared to SB polar bears.

Further support for our hypothesis that diet is a driver of intra-species differences in the polar bear gut microbiome comes from the results that focused on the SB polar bears, for which we determined dietary patterns using FA signatures. For these polar bears, the FA-PC scores explained the largest amount of the variance in gut bacteria alpha diversity of all explanatory variables considered. These FA-PC scores also explained large amounts of variation in gut bacterial composition at most bacterial taxonomic levels, particularly at the ASV-level. Other studies on wildlife have identified diet as an important long and short term driver of gut bacterial composition and diversity^8,9^, however the stable isotope diet analysis methods employed in previous wildlife work may not offer the resolution of dietary information relative to FAs^66^. Thus, our use of FA signatures to provide insight into variation in gut microbiome composition and diversity within and among wildlife populations shows considerable promise and suggest that future gut microbiome research could benefit from this approach. For example, quantitative fatty acid signature analysis (QFASA) and molecular-based diet analysis methods can provide species-level information on the diets of wild, free-ranging species which could enhance our understanding of how consumption of particular prey types influence the gut microbiome^67–69^.

In both humans and animals, sex/age class has been shown to impact gut bacterial composition and diversity^15,18,70,71^. Although the assumption of homogeneity of multivariate group dispersions for sex/age class groups was not met and we advise some caution in the interpretation of significant between-group differences, it is also possible that these differences in group dispersions could be related to important life history differences among polar bear sex/age classes. We found that bacteria from the class Negativicutes were higher in adult males compared to adult females, subadults, and cubs, while Saccharimonadia and Bacilli were higher in adult females and cubs compared to adult males and subadults. In addition to the probable health benefits of Bacilli discussed earlier, Bacilli may also be higher in females and cubs due to more *Lactobacillus* in the vaginal microbiome and in relation to milk production and lactation^58^, and other studies have detected higher levels of *Lactobacillus* in females compared to males as well^72^. Some human and mouse model studies have also demonstrated strong interactions between sex-specific hormones and commensal gut bacteria, which could also be driving a portion of the sex/age class differences observed here^73–75^. In addition, considering the varied foraging behavior among polar bear sex/age classes, these compositional differences likely have underlying associations with dietary differences just as for subpopulation and the two factors may also interact^21,76^. For example, adult male polar bears are much larger in body size and can more easily take down larger prey (bearded seal, beluga whale, etc.) when they are available, while adult females and subadults likely preferentially forage on smaller-bodied prey, such as ringed seal^30,77^. Additionally, for the SB subpopulation in particular, it has also been shown that adult male polar bears use bowhead whale ‘bone piles’ more frequently than other sex/age classes^78^, and consume higher amounts of bowhead whales compared to adult females^61^. Cubs of the year generally have a different diet entirely, as they rely on high-fat milk from their mothers. Further, we found lower Faith’s phylogenetic diversity in subadults compared to adult females which could be due to gut microbiomes of younger individuals being underdeveloped relative to adult microbiomes^15^. In general, diversity differences among sex/age classes likely reflect gut bacterial compositional differences that are tied to life history, physiological and diet differences among the sex/age classes.

We found no effect of body condition on variation in gut bacterial composition and diversity for SB and EG polar bears. It is possible that by choosing body mass as our indicator of body condition our results are confounded by other factors known to influence body mass of polar bears, such as sex and age class. However, to account for this, interaction terms were included in all models testing for associations between body condition and gut microbiota but none were found to be significant. While body condition has been identified as an important factor in some gut microbiome studies and potentially linked to diet^79^, other studies have similarly found minimal or no importance of BMI on gut microbiome composition and diversity^80,81^. Other biological and environmental factors could also contribute to differences in gut bacterial composition and diversity for EG and SB polar bears, including region-specific differences in contaminants, parasite types or loads, and differing interspecific interactions^29,65,82–84^. We were not able to account for these in our study, but such associations may be relevant to study in future work.

We found observable inter-individual variation within each subpopulation, which likely contributed to most (~ 85–90%) of the remaining unexplained variation in gut bacterial composition between the two subpopulations. However, it is important to note that other potential unmeasured biological factors and general stochasticity of the gut microbiome could also contribute to this unexplained variation^9,85^. Despite a greater number of total and unique ASVs within the SB relative to EG subpopulation, the lack of significant subpopulation differences in any of the alpha diversity indices measured could also reflect that high inter-individual variation is typical. Other studies have also noted a lack of intra-species or inter-population differences in alpha diversity, while still detecting significant compositional differences between groups^12,60^. Host phylogeny is another strong driver of gut bacterial composition and diversity and might, in part, explain the large overlap in bacterial species detected among EG and SB polar bears, the low separation between subpopulations along beta diversity NMDS axes, and the large amount of unexplained variation in gut bacterial composition and diversity^1,2^ as it has been suggested that gut microbiota are vertically transmitted and coevolve with their host species^86^. Additional metrics we were unable to account for in our study but that could be useful to include in future studies include cortisol levels as an indicator of stress-levels^87,88^, female reproductive status^89^, immune status of individuals by measuring cytokines^90,91^, assessment of individual contaminant loads^92–94^, etc. Many studies on the gut microbiome have also found high proportions of unexplained variation which can reflect the convoluted nature of microbiome data^2,85^. Given this typically is the case, and the fact that high inter-individual variation can sometimes mask generalized group differences, we can conclude there are relatively strong compositional differences in the gut bacteria for EG and SB polar bears.

Overall, this study showed differences in gut composition and diversity between two geographically distant polar bear subpopulations facing distinct sea ice conditions and prey availability. The SB subpopulation showed more rare and unique ASVs and bacterial genera present compared to the EG subpopulation and indications of greater inter-individual diversity. These findings likely, in part, reflect the use of onshore foods for some members of the population during the reduced ice season^62,95^. This interpretation is supported by the SB subset results indicating diet and intraspecific variation among polar bear sex/age classes are likely linked, and are key drivers of alpha diversity and gut bacterial composition within the subpopulation, This study highlights the importance of considering both inter-population and inter-individual variation in gut bacterial composition, given the direct links between gut microbiota and host physiology, nutrition, and overall health^96,97^. Nonetheless, because there are many variables that influence the gut bacterial community, it can be challenging to assess the influence of each in isolation, or to make direct conclusions when certain factors are unavailable for assessment. Polar bears are facing a myriad of anthropogenic stressors posing threats to their continued survival as a species. Moving forward, assessing the impacts of such stressors on the gut microbiome will likely be an important aspect of monitoring polar bear health.

## Materials and methods

### Collection of polar bear fecal and adipose tissue

Fecal samples were collected from 34 EG polar bears in March–April of 2017 and from 59 SB polar bears in March–April of 2015, 2016, 2018, and 2019 (Fig. 1). Polar bears were immobilized from a helicopter and tissue samples were collected as part of long-term population assessments in each region. Biometric measurements were recorded, including sex and body mass. Ages were quantitatively estimated via growth layer groups from a vestigial premolar tooth sampled on first capture^98^. Fecal samples were collected from the rectum of polar bears using sterile latex gloves placed in sterile whirlpak bags. Due to limitations imposed by the COVID-19 pandemic, only adipose tissue samples from SB polar bears could be shipped and analyzed for fatty acid-based assessment of diet. Adipose tissue biopsies were collected from 46 SB polar bears, representing a subset of the same SB individuals for which fecal samples were taken. Fecal and adipose samples were kept at -20 °C during the field season and then shipped on dry ice to McGill and stored at -80 °C prior to laboratory analysis. Samples were collected from SB polar bears as part of the U.S. Geological Survey (USGS) Polar Bear Research Program (U.S. Fish and Wildlife Service Permit# MA690038 to T.C.A) under capture protocols approved by the USGS Institutional Animal Care and Use Committee. Samples were collected from EG polar bears under case nr. 2017-5446, document 4710596 from the Department of Fisheries and Hunting, as part of a long-term monitoring program by the Greenland Institute of Natural Resources.

### Fecal DNA extraction

Fecal samples were extracted in random order at McGill University according to the same procedures previously described for samples from 2009 to 2013 from the SB polar bear subpopulation^29^. Briefly, feces from the glove were swabbed with a sterile cotton-tipped applicator. Tips were transferred to a tube of 1 mL phosphate-buffered saline (PBS), vortexed, and spun down after removing the cotton tip to obtain a pellet. After adding a stainless-steel bead (Qiagen; Hilden, Germany) and lysis buffer (see Watson et al.^29^), samples were homogenized at 37 °C in a shaking water bath. The extraction protocol then continued at step 2 of the QIAamp Mini Kit Buccal Swab Spin Protocol (QIAamp DNA Mini and Blood Mini Handbook). Samples were spun down in a final volume of 100 μL elution buffer (Buffer AE) and 30 μL of each extract was aliquoted among two 96-well plates to facilitate downstream PCR reaction setup. For each batch of extractions, a separate sterile swab control was run alongside samples as a blank and stored with corresponding samples on the same 96-well plate. All DNA extracts were stored at −20 °C until further analysis.

### 16S rRNA gene amplification and sequencing

Gene amplification was performed as per previous analyses on SB polar bears^29^ with minor modifications. In brief, a ~ 460 base pair (bp) region of the 16S rRNA gene was amplified using the universal bacterial primer set 341F (5′-CCTACGG GNGGCWGCAG-3′) and 805Rmod (5’-GACTACNVGGGTWTCTAATCC-3’) with overhanging Illumina adaptors. PCR reaction wells contained 6.5 μL of Rnase free H_2_O, 0.5 μL of 20 mg mL^−1^ BSA (bovine serum albumin), 1.5 μL of 10 μg μL^−1^ of both 341F and 805Rmod primers, 12.5 μL of 2X Kapa Hifi Hot Start Ready Mix (Roche Diagnostics), and 2.5 μL template DNA with PCR cycling conditions as described^29^. Amplified DNA was purified using AMPure beads (0.8 bead to sample ratio; Beckman Coulter, Brea, CA, USA) according to the manufacturer’s instructions. Illumina® Nextera XT indices and sequencing adaptors (Illumina®, San Diego, CA) were annealed to PCR product in a subsequent 8-cycle PCR run as specified in the Illumina® 16S Library Preparation guide and purified again using AMPure beads (1.12 bead to sample ratio). Final indexed samples and negative controls were quantified using a Qubit fluorometer (Invitrogen, Thermo Fisher Scientific, USA) and pooled at 4 nM to create the final library, which was then characterized and validated using the Agilent 2100 Bioanalyzer (Agilent Technologies) confirming uniform amplicon size (~ 600 bp) before sequencing on a 2 × 250 bp paired-end run with v2 chemistry on an Illumina® MiSeq platform at McGill University.

### Fatty acid analysis

The 46 SB adipose tissue biopsies were processed for FA signatures to provide insight into feeding patterns according to methods previously used for SB polar bears from 2004 to 2016^61,69^. In short, lipids were extracted and then FAs were converted to fatty acid methyl esters (FAMEs) using the Hilditch reagent. FAMEs within each sample were then separated and analyzed on an Agilent (Santa Clara, CA, USA) 8860 gas chromatograph with flame ionization detector and quantified using OpenLab CDS Data Analysis software (V. 2.5; Agilent, Santa Clara, CA, USA) as mass percent of total FAME. FAs were abbreviated according to their carbon chain length (A), number of double bonds (B), and position of the first double bond counting from the methyl end of the carbon chain (X) as A:BnX.

### FA signatures as dietary indicators

A principal components analysis (PCA) was conducted using selected FAs to reduce dimensionality of the diet data, and the significant PC axes were then used as explanatory variables in PERMANOVA and multiple linear regression models explaining variation in gut bacterial composition and diversity. Of the 70 marine-associated FAs that were detected and quantified, 30 FAs thought to be present in polar bear adipose tissue predominately due to dietary uptake and used in previous polar bear diet studies were initially selected^21,99^. We did not include 20:1n11, as it has recently been suggested that this FA may not be informative in delineating polar bear feeding patterns^100^. We further only included the major dietary FAs, or those comprising on average > 1% of total FAME, to reduce the possible influence on FA proportions related to instrumental analytical variation^101^. The final set of nine FAs allowed us to meet the recommended 5:1 sample to variable ratio for conducting PCA analysis^102^. Prior to PCA analysis, the FA proportions were log-ratio transformed as recommended to normalize the multivariate data^102,103^.

### Microbial data analyses

Unless stated otherwise, all analyses were performed using R 4.0.3^104^. Sequencing data was filtered, trimmed, de-replicated, and paired ends were merged using DADA2^105^. The inferred amplicon sequence variants (ASVs) were taxonomically assigned using the SILVA reference database (version 132) as described in the DADA2 tutorial. Decontam^106^ was used to identify and filter out any contaminant ASVs, (i.e., those detected in both sample PCR negative controls and in extraction kit blanks). MicrobiomeAnalystR^107^ was then used to remove ASVs with less than 2 counts and zero variance and the resulting phyloseq object output was extracted and integrated into subsequent phyloseq^108^ and MicrobiomeAnalystR workflows^107,108^. All samples produced > 10,000 reads and so none were eliminated. As recommended, data rarefaction was not performed^109^.

MicrobiomeAnalyst was used to visually compare gut microbial composition between EG and SB polar bears at varying bacterial taxonomic levels. Shannon, Inverse Simpson, and Faith’s phylogenetic alpha diversity indices were calculated separately for EG and SB polar bears at ASV-level and subsequently using MicrobiomeAnalyst Web version (as per Watson et al.^29^). To provide insight into the biological and ecological variables responsible for differences in bacterial community composition (at bacterial phylum, class, genus, and ASV-level) within and among the EG and SB polar bears, permutational multivariate analysis of variance (PERMANOVA) tests were performed using the ‘adonis’ function in the vegan package in R^110^. The Bray–Curtis distance method was used for all bacterial taxonomic levels to assess compositional patterns at multiple levels, and Weighted UniFrac distance was used at just the ASV-level to incorporate the influence of bacterial phylogeny in our community composition comparisons. Homogeneity of group dispersions (PERMDISP^111^) for compared groups was checked prior to interpretation of PERMANOVA results. Subsequent analysis of composition with bias correction (ANCOMBC^112^) tests were done to compare differential abundances of specific bacterial classes, genera, and ASVs contributing to compositional differences.

Both multiple linear regression models and PERMANOVAs were used to test for other ecological effects on alpha diversity indices as well as compositional differences (i.e., beta diversity differences). For both the PERMANOVAs and the linear models (LMs), the additional explanatory variables included subpopulation, sex/age class, body mass (as an indicator of body condition;^113^), and all biologically-relevant first-order interactions. The sex/age classes used were adult female (AF, *n* = 36), adult male (AM, *n* = 32), subadult (S, *n* = 15), and cub (C, *n* = 10). Year of capture was not included as an explanatory variable as EG bears were only captured in a single year and years did not overlap for the two subpopulations. When categorical explanatory variables were found to be significant in the PERMANOVAs or LMs, *post-hoc* univariate tests (ANOVAs) and ANCOMBC tests were performed to determine which means and bacterial classes, genera, and ASVs significantly differed between groups (e.g., sex/age classes).

Given that we only had FA signatures for SB polar bears, separate PERMANOVAs using the Bray–Curtis distance method (and *post-hoc* univariate tests, as appropriate) were also run to examine associations of bacterial composition with diet, using the significant PCs from the FA analysis (as described above), while also including sex/age class, body mass, and capture year (2016, 2017, and 2018). The sex/age classes used were adult female (AF, *n* = 16), adult male (AM, *n* = 24), and subadult (S, *n* = 6). Cubs are not included as adipose biopsies were not collected from this age class. Multiple linear regression models were run to test for ecological and dietary effects on gut bacterial alpha and beta diversity (represented by Bray–Curtis and Weighted UniFrac NMDS axes) indices for SB polar bears. Top models were selected using backwards model selection and Akaike information criterion (*AIC*) scores adjusted for smaller sample sizes^114^. The backwards model selection process was conducted via step-wise dropping of terms in the model and AIC calculation. If dropping a term decreased the AIC it was removed and this process repeated until removal of variables did not result in lowering of the AIC score of the overall model.

## Supplementary Information

Supplementary Information.

## Data Availability

The 16S sequencing data have been deposited in the NCBI Short Read Archive under project number PRJNA773176. The fatty acid data are available as a Supporting Information file.
