# Supplementary material for: Distinct gut microbiomes in two polar bear subpopulations inhabiting different sea ice ecoregions
Source: Sci Rep. 2022 Jan 11;12:522. doi: 10.1038/s41598-021-04340-2 (PMC8752607; doi:10.1038/s41598-021-04340-2)
Supplement: Supplementary file 1 — Supplementary Information. [file 41598_2021_4340_MOESM1_ESM.docx]

**Supplementary Information:**

**Supplementary Table S1.** Summary of linear regression models showing that none of the explanatory variables explained a significant amount of the variation in Shannon and Inverse Simpson indices of alpha diversity. There was a tendency towards a significant effect of sex/age class in linear regression model for Faith’s phylogenetic diversity (FPD), suggesting lower FPD in subadults compared to other sex/age classes. SB = Southern Beaufort Sea.

| **Shannon Diversity** (Adj. R^2^ = 0.00, *p* = 0.67) | | | | | |
| --- | --- | --- | --- | --- | --- |
| Full Model: | ~ subpopulation + sex/age class + body condition + sex/age class*subpopulation + body condition*subpopulation | | | | |
|  | Coefficients | Estimate | Std.Error | tvalue | Pr(>\|t\|) |
|  | (Intercept) | 2.52 | 0.33 | 7.72 | <0.001 |
|  | Subpopulation (SB) | 0.27 | 0.43 | 0.62 | 0.53 |
|  | Sex/age class (Adult male) | 0.02 | 0.56 | 0.035 | 0.97 |
|  | Sex/age class (Cub) | 0.10 | 0.27 | 0.39 | 0.70 |
|  | Sex/age class (Subadult) | -0.03 | 0.25 | -0.12 | 0.91 |
|  | Body Condition | 0.0005 | 0.002 | 0.29 | 0.77 |
|  | Subpopulation (SB): Sex/age class (Adult male) | -0.09 | 0.63 | -0.15 | 0.88 |
|  | Subpopulation (SB): Sex/age class (Cub) | -0.06 | 0.40 | -0.15 | 0.88 |
|  | Subpopulation (SB): Sex/age class (Subadult) | -0.36 | 0.32 | -1.12 | 0.27 |
|  | Subpopulation (SB): Body Condition | -0.0003 | 0.002 | -0.15 | 0.88 |
| **Inverse Simpson Diversity** (Adj. R^2^ = 0.00, *p* = 0.74) | | | | | |
| Full Model: | ~ subpopulation + sex/age class + body condition + sex/age class*subpopulation + body condition*subpopulation | | | | |
|  | Coefficients | Estimate | Std.Error | tvalue | Pr(>\|t\|) |
|  | (Intercept) | 5.63 | 2.75 | 2.05 | 0.044 |
|  | Subpopulation (SB) | 3.30 | 3.59 | 0.92 | 0.36 |
|  | Sex/age class (Adult male) | -1.97 | 4.72 | -0.42 | 0.68 |
|  | Sex/age class (Cub) | 0.85 | 2.24 | 0.38 | 0.71 |
|  | Sex/age class (Subadult) | -0.40 | 2.12 | -0.19 | 0.85 |
|  | Body Condition | 0.014 | 0.015 | 0.93 | 0.35 |
|  | Subpopulation (SB): Sex/age class (Adult male) | 1.44 | 5.27 | 0.27 | 0.78 |
|  | Subpopulation (SB): Sex/age class (Cub) | -0.11 | 3.37 | -0.032 | 0.97 |
|  | Subpopulation (SB): Sex/age class (Subadult) | -1.67 | 2.67 | -0.63 | 0.53 |
|  | Subpopulation (SB): Body Condition | -0.011 | 0.018 | -0.62 | 0.54 |
| **Faiths Phylogenetic Diversity** (Adj. R^2^ = 0.06, *p* = 0.12) | | | | | |
| Full Model: | ~ subpopulation + sex/age class+ body condition + sex/age class*subpopulation + body condition*subpopulation | | | | |
|  | Coefficients | Estimate | Std.Error | tvalue | Pr(>\|t\|) |
|  | (Intercept) | 13.64 | 1.80 | 7.58 | 4.4E-11 |
|  | Subpopulation (SB) | 1.13 | 2.36 | 0.48 | 0.63 |
|  | Sex/age class (Adult male) | 1.18 | 3.09 | 0.38 | 0.70 |
|  | Sex/age class (Cub) | -0.29 | 1.47 | -0.19 | 0.85 |
|  | **Sex/age class (Subadult)** | **-2.49** | **1.39** | **-1.78** | **0.078** |
|  | Body Condition | -0.0028 | 0.0096 | -0.29 | 0.77 |
|  | Subpopulation (SB): Sex/age class (Adult male) | -3.30 | 3.45 | -0.96 | 0.34 |
|  | Subpopulation (SB): Sex/age class (Cub) | -1.32 | 2.21 | -0.60 | 0.55 |
|  | Subpopulation (SB): Sex/age class (Subadult) | -0.42 | 1.75 | -0.24 | 0.81 |
|  | Subpopulation (SB): Body Condition | 0.0029 | 0.012 | 0.25 | 0.80 |

**Supplementary Table S2**. Results of Analysis of Composition with bias correction (ANCOM-BC) analysis two classes of bacteria that differed in abundance between East Greenland (EG) and Southern Beaufort Sea (SB) polar bear subpopulations (False discovery rate (FDR) adjusted *p*-value cutoff: 0.05). Coef. = log-transformed change in abundance, SE = standard error of the coefficient, W = Coef./SE.

|  | Class | Coef.  (SB - EG) | *SE* | *Test statistic (W)* | *p-value* | *Adj. p-value* |
| --- | --- | --- | --- | --- | --- | --- |
| 1 | Bacilli | -1.88 | 0.42 | -4.49 | <0.001 | <0.001 |
| 2 | Coriobacteriia | -0.95 | 0.32 | -2.92 | 0.003 | 0.045 |

**Supplementary Table S3**. Results of Analysis of Composition with bias correction (ANCOM-BC) analysis (for the 21 bacterial genera that differed in abundance between East Greenland (EG) and Southern Beaufort Sea (SB) polar bear subpopulations (False discovery rate (FDR) adjusted *p*-value cutoff: 0.05). Coef. = log-transformed change in abundance, SE = standard error of the coefficient, W = Coef./SE. Asterisks (*) indicate differentially abundant genera that are also comprise a portion of the top ~90% of reads obtained for EG and SB polar bears combined.

|  | Genus | Coef.  (SB vs. EG) | *SE* | *Test statistic (W)* | *p-value* | *Adj. P-value* |
| --- | --- | --- | --- | --- | --- | --- |
| 1 | **Anaerococcus* | 3.16 | 0.55 | 5.71 | <0.001 | <0.001 |
| 2 | **Murdochiella* | 3.07 | 0.52 | 5.96 | <0.001 | <0.001 |
| 3 | **Megasphaera* | 2.70 | 0.72 | 3.76 | <0.001 | 0.014 |
| 4 | *Dialister* | 1.89 | 0.50 | 3.81 | <0.001 | 0.012 |
| 5 | *Anaerobiospirillum* | 1.68 | 0.43 | 3.94 | <0.001 | 0.007 |
| 6 | *Anoxybacillus* | 1.50 | 0.38 | 3.93 | <0.001 | <0.001 |
| 7 | *Hydrogenophilus* | 1.29 | 0.33 | 3.94 | <0.001 | <0.001 |
| 8 | *Parabacteroides* | 0.60 | 0.28 | 2.15 | <0.001 | <0.001 |
| 9 | *Anaerostipes* | 0.46 | 0.26 | 1.79 | <0.001 | <0.001 |
| 10 | *Cloacibacterium* | 0.12 | 0.23 | 0.50 | <0.001 | <0.001 |
| 11 | *Parvimonas* | -1.66 | 0.44 | -3.77 | <0.001 | 0.014 |
| 12 | *Rothia* | -1.69 | 0.45 | -3.78 | <0.001 | 0.013 |
| 13 | *TM7x* | -1.70 | 0.47 | -3.64 | <0.001 | 0.023 |
| 14 | *Brachybacterium* | -1.78 | 0.42 | -4.27 | <0.001 | 0.002 |
| 15 | *Leucobacter* | -1.80 | 0.52 | -3.50 | <0.001 | 0.037 |
| 16 | **Escherichia_Shigella* | -1.87 | 0.43 | -4.35 | <0.001 | 0.001 |
| 17 | **Collinsella* | -1.98 | 0.43 | -4.59 | <0.001 | <0.001 |
| 18 | *Turicibacter* | -2.29 | 0.62 | -3.72 | <0.001 | 0.017 |
| 19 | *Enterococcus* | -2.42 | 0.57 | -4.28 | <0.001 | 0.002 |
| 20 | **Erysipelatoclostridium* | -2.88 | 0.55 | -5.28 | <0.001 | <0.001 |
| 21 | **Lactobacillus* | -3.66 | 0.72 | -5.07 | <0.001 | <0.001 |

**Supplementary Table S4**. Results of Analysis of Composition with bias correction (ANCOM-BC) analysis for the 48 amplicon sequence variants (ASVs) that significantly differed in abundance between East Greenland (EG) and Southern Beaufort Sea (SB) polar bear subpopulations (False discovery rate (FDR) adjusted *p*-value cutoff: 0.05). Coef. = log-transformed change in abundance, SE = standard error of the coefficient, W = Coef./SE.

|  | ASV | Class | Genus | Coefficient  (SB vs. EG) | *SE.* | *Test statistic (W)* | *p-value* | *Adj. p-value* |
| --- | --- | --- | --- | --- | --- | --- | --- | --- |
| 1 | ASV_49 | Negativicutes | *Dialister* | 3.54 | 0.41 | 8.59 | <0.001 | <0.001 |
| 2 | ASV_20 | Clostridia | *Anaerococcus* | 2.76 | 0.57 | 4.81 | <0.001 | <0.001 |
| 3 | ASV_26 | Clostridia | *Murdochiella* | 2.58 | 0.48 | 5.34 | <0.001 | <0.001 |
| 4 | ASV_92 | Clostridia | *Peptoniphilus* | 2.06 | 0.37 | 5.57 | <0.001 | <0.001 |
| 5 | ASV_46 | Bacteroidia | *Porphyromonas* | 2.00 | 0.44 | 4.51 | <0.001 | 0.002 |
| 6 | ASV_52 | Negativicutes | *Megasphaera* | 1.97 | 0.45 | 4.35 | <0.001 | <0.001 |
| 7 | ASV_105 | Clostridia | *Fastidiosipila* | 1.78 | 0.37 | 4.87 | <0.001 | <0.001 |
| 8 | ASV_72 | Gammaproteobacteria | *Anaerobiospirillum* | 1.73 | 0.39 | 4.46 | <0.001 | 0.002 |
| 9 | ASV_43 | Clostridia | *Murdochiella* | 1.45 | 0.43 | 3.39 | <0.001 | <0.001 |
| 10 | ASV_136 | Bacilli | *Anoxybacillus* | 1.29 | 0.36 | 3.62 | <0.001 | <0.001 |
| 11 | ASV_173 | Gammaproteobacteria | *Hydrogenophilus* | 1.22 | 0.31 | 3.90 | <0.001 | <0.001 |
| 12 | ASV_120 | Clostridia | *Helococcus* | 1.21 | 0.37 | 3.31 | <0.001 | <0.001 |
| 13 | ASV_121 | Coriobacteriia | *Olsenella* | 0.80 | 0.34 | 2.33 | <0.001 | <0.001 |
| 14 | ASV_266 | Campylobacteria | *Campylobacter* | 0.75 | 0.29 | 2.57 | <0.001 | <0.001 |
| 15 | ASV_159 | Negativicutes | *Megasphaera* | 0.73 | 0.34 | 2.12 | <0.001 | <0.001 |
| 16 | ASV_363 | Bacilli | *Anoxybacillus* | 0.53 | 0.26 | 2.03 | <0.001 | <0.001 |
| 17 | ASV_274 | Clostridia | *NA* | 0.50 | 0.27 | 1.83 | <0.001 | <0.001 |
| 18 | ASV_252 | Clostridia | *Peptostreptococcus* | 0.47 | 0.30 | 1.53 | <0.001 | <0.001 |
| 19 | ASV_277 | Bacteroidia | *Bacteroides* | 0.42 | 0.28 | 1.48 | <0.001 | <0.001 |
| 20 | ASV_491 | Clostridia | *Anaerostipes* | 0.40 | 0.24 | 1.67 | <0.001 | <0.001 |
| 21 | ASV_361 | Bacteroidia | *Parabacteroides* | 0.37 | 0.26 | 1.43 | <0.001 | <0.001 |
| 22 | ASV_1799 | Bacteroidia | *Bacteroides* | -0.70 | 0.18 | -3.81 | <0.001 | 0.029 |
| 23 | ASV_978 | Bacilli | *NA* | -0.99 | 0.25 | -4.00 | <0.001 | 0.014 |
| 24 | ASV_1004 | Bacilli | *Staphylococcus* | -1.09 | 0.27 | -3.99 | <0.001 | 0.015 |
| 25 | ASV_525 | Saccharimonadia | *TM7x* | -1.28 | 0.34 | -3.78 | <0.001 | 0.034 |
| 26 | ASV_294 | Bacilli | *Streptococcus* | -1.60 | 0.40 | -3.96 | <0.001 | 0.017 |
| 27 | ASV_279 | Clostridia | *Parvimonas* | -1.60 | 0.42 | -3.81 | <0.001 | 0.029 |
| 28 | ASV_157 | Actinobacteria | *Brachybacterium* | -1.84 | 0.42 | -4.34 | <0.001 | 0.003 |
| 29 | ASV_312 | Campylobacteria | *Campylobacter* | -1.85 | 0.41 | -4.47 | <0.001 | 0.002 |
| 30 | ASV_356 | Bacilli | *NA* | -1.91 | 0.37 | -5.15 | <0.001 | <0.001 |
| 31 | ASV_1 | Gammaproteobacteria | *Escherichia/Shigella* | -1.91 | 0.41 | -4.69 | <0.001 | <0.001 |
| 32 | ASV_99 | Bacilli | *Gemella* | -1.95 | 0.53 | -3.71 | <0.001 | 0.044 |
| 33 | ASV_80 | Gammaproteobacteria | *Klebsiella* | -1.98 | 0.52 | -3.82 | <0.001 | 0.028 |
| 34 | ASV_160 | Negativicutes | *Dialister* | -2.01 | 0.46 | -4.36 | <0.001 | 0.003 |
| 35 | ASV_6 | Coriobacteriia | *Collinsella* | -2.10 | 0.42 | -4.94 | <0.001 | <0.001 |
| 36 | ASV_115 | Actinobacteria | *Actinomyces* | -2.16 | 0.51 | -4.20 | <0.001 | 0.006 |
| 37 | ASV_24 | Clostridia | *Lachnoclostridium* | -2.25 | 0.58 | -3.92 | <0.001 | 0.020 |
| 38 | ASV_23 | Gammaproteobacteria | *Klebsiella* | -2.35 | 0.62 | -3.83 | <0.001 | 0.028 |
| 39 | ASV_39 | Bacilli | *Turicibacter* | -2.36 | 0.60 | -3.93 | <0.001 | 0.018 |
| 40 | ASV_60 | Clostridia | *Clostridium_sensu_stricto_1* | -2.38 | 0.58 | -4.09 | <0.001 | 0.010 |
| 41 | ASV_36 | Clostridia | *Terrisporobacter* | -2.41 | 0.62 | -3.91 | <0.001 | 0.021 |
| 42 | ASV_208 | Actinobacteria | *Corynebacterium* | -2.47 | 0.47 | -5.27 | <0.001 | <0.001 |
| 43 | ASV_59 | Bacilli | *Enterococcus* | -2.68 | 0.54 | -4.99 | <0.001 | <0.001 |
| 44 | ASV_16 | Clostridia | *Blautia* | -2.82 | 0.68 | -4.14 | <0.001 | 0.008 |
| 45 | ASV_62 | Bacilli | *Erysipelatoclostridium* | -2.91 | 0.53 | -5.55 | <0.001 | <0.001 |
| 46 | ASV_9 | Bacilli | *Streptococcus* | -2.94 | 0.77 | -3.82 | <0.001 | 0.029 |
| 47 | ASV_14 | Negativicutes | *Megamonas* | -2.97 | 0.71 | -4.21 | <0.001 | 0.006 |
| 48 | ASV_8 | Bacilli | *Lactobacillus* | -3.77 | 0.70 | -5.39 | <0.001 | <0.001 |

**Supplementary Table S5**. Results of Analysis of Composition with bias correction (ANCOM-BC) analysis for the three classes of bacteria that differed in abundance among sex/age classes (adult females [AF], adult males [AM] and subadults [S] compared to cubs [C]) for East Greenland (EG) and Southern Beaufort Sea (SB) polar bear subpopulations (False discovery rate (FDR) adjusted *p*-value cutoff: 0.05).

|  | Class | Coef.  AF-C | Coef.  AM-C | Coef.  S - C | SE  AF-C | SE  AM - C | SE  S - C | W  Statistic  AF - C | W  Statistic  AM - C | W  Statistic  S - C | *p -*value AF-C | *p* -value  AM - C | *p* -value S - C | FDR Adj.  *p* -value  AF - C | FDR Adj.  *p* -value  AM - C | FDR  Adj.  *p* -value  S - C |
| --- | --- | --- | --- | --- | --- | --- | --- | --- | --- | --- | --- | --- | --- | --- | --- | --- |
| 1 | Bacilli | -0.973 | -2.32 | -1.21 | 0.601 | 0.585 | 0.763 | -1.62 | -3.97 | -1.58 | 0.105 | 0.00 | 0.113 | 1.00 | 0.001 | 1.00 |
| 2 | Parcubacteria | 0.059 | 0.379 | 0.378 | 0.448 | 0.473 | 0.491 | 0.131 | 0.802 | 0.771 | 0.00 | 0.00 | 0.00 | 0.00 | 0.00 | 0.00 |
| 3 | Saccharimonadia | -0.424 | -2.42 | -0.915 | 0.845 | 0.797 | 0.918 | -0.502 | -3.03 | -0.997 | 0.616 | 0.002 | 0.319 | 1.00 | 0.032 | 1.00 |

**Supplementary Table S6**. Results of Analysis of Composition with bias correction (ANCOM-BC) analysis for the 21 bacterial genera that significantly differed in abundance sex/age classes (adult females [AF], adult males [AM] and subadults [S] compared to cubs [C]) for East Greenland (EG) and Southern Beaufort Sea (SB) polar bear subpopulations (False discovery rate (FDR) adjusted *p*-value cutoff: 0.05)

| Genus | Coef.  AF-C | Coef.  AM-C | Coef.  S - C | SE  AF-C | SE  AM - C | SE  S - C | W  Statistic  AF - C | W  Statistic  AM - C | W  Statistic  S - C | *p -*value AF-C | *p* -value  AM - C | *p -*value  S - C | FDR Adj.  *p* -value  AF - C | FDR  Adj.  *p* -value  AM - C | FDR  Adj.  *p* -value  S - C |
| --- | --- | --- | --- | --- | --- | --- | --- | --- | --- | --- | --- | --- | --- | --- | --- |
| *Trueperella* | 1.600 | 3.596 | -0.328 | 0.688 | 0.814 | 0.780 | 2.326 | 4.416 | -0.421 | 0.020 | 0.000 | 0.674 | 1.000 | 0.001 | 1.000 |
| *Arthrobacter* | 0.428 | 0.004 | 0.365 | 0.524 | 0.510 | 0.685 | 0.816 | 0.007 | 0.533 | 0.000 | 0.000 | 0.000 | 0.000 | 0.000 | 0.000 |
| *Parabacteroides* | 0.178 | 1.515 | 0.459 | 0.312 | 0.459 | 0.459 | 0.571 | 3.302 | 1.002 | 0.000 | 0.000 | 0.000 | 0.000 | 0.000 | 0.000 |
| *Ignavigranum* | -0.119 | -1.254 | -1.520 | 0.928 | 0.946 | 0.896 | -0.128 | -1.326 | -1.696 | 0.000 | 0.000 | 0.000 | 0.000 | 0.000 | 0.000 |
| *Clostridium_sensu_stricto_7* | 0.538 | 1.924 | 0.944 | 0.456 | 0.539 | 0.586 | 1.181 | 3.571 | 1.611 | 0.000 | 0.000 | 0.000 | 0.000 | 0.000 | 0.000 |
| *Hathewaya* | 0.085 | 1.336 | 0.412 | 0.327 | 0.530 | 0.450 | 0.259 | 2.520 | 0.915 | 0.000 | 0.000 | 0.000 | 0.000 | 0.000 | 0.000 |
| *Tuzzerella* | 0.170 | 0.965 | 0.993 | 0.361 | 0.378 | 0.552 | 0.470 | 2.553 | 1.799 | 0.000 | 0.000 | 0.000 | 0.000 | 0.000 | 0.000 |
| *UBA1819* | -0.170 | 1.335 | 0.400 | 0.291 | 0.445 | 0.410 | -0.585 | 3.004 | 0.976 | 0.000 | 0.000 | 0.000 | 0.000 | 0.000 | 0.000 |
| *Peptostreptococcus* | -0.796 | 2.542 | -0.445 | 0.692 | 0.723 | 1.014 | -1.150 | 3.516 | -0.439 | 0.250 | 0.000 | 0.661 | 1.000 | 0.040 | 1.000 |
| *Anaerococcus* | 2.075 | 4.760 | 0.952 | 0.640 | 0.824 | 0.710 | 3.241 | 5.773 | 1.341 | 0.001 | 0.000 | 0.180 | 0.117 | 0.000 | 1.000 |
| *Ezakiella* | -2.949 | -3.921 | -2.815 | 0.957 | 0.965 | 1.142 | -3.080 | -4.062 | -2.464 | 0.002 | 0.000 | 0.014 | 0.200 | 0.005 | 1.000 |
| *Gallicola* | -0.717 | -0.830 | -1.119 | 0.802 | 0.828 | 0.777 | -0.893 | -1.003 | -1.440 | 0.000 | 0.000 | 0.000 | 0.000 | 0.000 | 0.000 |
| *Guggenheimella* | 0.173 | 0.109 | 0.480 | 0.404 | 0.429 | 0.559 | 0.428 | 0.254 | 0.859 | 0.000 | 0.000 | 0.000 | 0.000 | 0.000 | 0.000 |
| *Murdochiella* | 0.681 | 3.893 | -0.208 | 0.794 | 0.952 | 0.792 | 0.858 | 4.090 | -0.263 | 0.391 | 0.000 | 0.793 | 1.000 | 0.004 | 1.000 |
| *Soehngenia* | 0.075 | -0.399 | -0.189 | 0.488 | 0.512 | 0.591 | 0.154 | -0.779 | -0.320 | 0.000 | 0.000 | 0.000 | 0.000 | 0.000 | 0.000 |
| *W5053* | 0.287 | -0.097 | 0.217 | 0.468 | 0.483 | 0.562 | 0.613 | -0.202 | 0.387 | 0.000 | 0.000 | 0.000 | 0.000 | 0.000 | 0.000 |
| *Dialister* | 1.003 | 3.416 | -0.052 | 0.734 | 0.776 | 0.890 | 1.366 | 4.405 | -0.059 | 0.172 | 0.000 | 0.953 | 1.000 | 0.001 | 1.000 |
| *Methylorubrum* | 0.180 | 0.968 | 0.728 | 0.296 | 0.398 | 0.457 | 0.607 | 2.433 | 1.595 | 0.000 | 0.000 | 0.000 | 0.000 | 0.000 | 0.000 |
| *Enterobacter* | 0.108 | 1.049 | 0.157 | 0.334 | 0.444 | 0.305 | 0.324 | 2.365 | 0.513 | 0.000 | 0.000 | 0.000 | 0.000 | 0.000 | 0.000 |
| *Moraxella* | -0.056 | 1.134 | 0.269 | 0.293 | 0.386 | 0.334 | -0.191 | 2.940 | 0.805 | 0.000 | 0.000 | 0.000 | 0.000 | 0.000 | 0.000 |
| *Stenotrophomonas* | -0.568 | 0.546 | -0.368 | 0.463 | 0.584 | 0.466 | -1.227 | 0.935 | -0.790 | 0.000 | 0.000 | 0.000 | 0.000 | 0.000 | 0.000 |

**Supplementary Table S7**.

Results of Analysis of Composition with bias correction (ANCOM-BC) analysis for the 78 amplicon sequence variants (ASVs) that significantly differed in abundance among polar bear sex/age classes (adult females [AF], adult males [AM] and subadults [S] compared to cubs [C]) for East Greenland (EG) and Southern Beaufort Sea (SB) polar bear subpopulations (False discovery rate (FDR) adjusted *p*-value cutoff: 0.05)

| **ASV** | **Bacterial Class** | **Bacterial Genus** | **Coef.**  **(AF - C)** | **Coef.**  **(AM - C)** | **Coef.**  **(S - C)** | **SE**  **(AF - C)** | **SE**  **(AM - C)** | **SE**  **(S - C)** | **Test statistic [W]**  **(AF - C)** | **Test statistic [W]**  **(AM - C)** | **Test statistic [W]**  **(S - C)** | ***p -*value**  **(AF - C)** | ***p -*value**  **(AM - C)** | ***p -*value**  **(S - C)** | **FDR Adj. *p* -value**  **(AF - C)** | **FDR Adj. *p* -value**  **(AM - C)** | **FDR Adj. *p* -value**  **(S - C)** |
| --- | --- | --- | --- | --- | --- | --- | --- | --- | --- | --- | --- | --- | --- | --- | --- | --- | --- |
| ASV_15 | Clostridia | *Peptostreptococcus* | 0.102 | 3.085 | 0.189 | 0.850 | 0.830 | 1.135 | 0.120 | 3.718 | 0.166 | 0.905 | 0.000 | 0.868 | 1.000 | 0.049 | 1.000 |
| ASV_20 | Clostridia | *Anaerococcus* | 1.875 | 4.529 | 0.736 | 0.555 | 0.693 | 0.500 | 3.379 | 6.540 | 1.473 | 0.000 | 0.000 | 0.000 | 0.000 | 0.000 | 0.000 |
| ASV_22 | Clostridia | *Fastidiosipila* | 3.383 | -0.108 | 1.140 | 0.697 | 0.545 | 0.906 | 4.854 | -0.198 | 1.259 | 0.000 | 0.843 | 0.208 | 0.000 | 1.000 | 1.000 |
| ASV_26 | Clostridia | *Murdochiella* | 0.815 | 3.382 | 0.148 | 0.572 | 0.721 | 0.588 | 1.427 | 4.689 | 0.251 | 0.154 | 0.000 | 0.801 | 1.000 | 0.001 | 1.000 |
| ASV_29 | Clostridia | *Ezakiella* | -2.941 | -4.433 | -2.846 | 0.981 | 0.937 | 1.157 | -2.999 | -4.733 | -2.460 | 0.003 | 0.000 | 0.014 | 0.665 | 0.001 | 1.000 |
| ASV_41 | Actinobacteria | *Trueperella* | 1.536 | 3.928 | 0.220 | 0.533 | 0.597 | 0.552 | 2.883 | 6.577 | 0.398 | 0.004 | 0.000 | 0.691 | 0.955 | 0.000 | 1.000 |
| ASV_43 | Clostridia | *Murdochiella* | 0.093 | 1.575 | -0.560 | 0.718 | 0.817 | 0.588 | 0.130 | 1.927 | -0.952 | 0.000 | 0.000 | 0.000 | 0.000 | 0.000 | 0.000 |
| ASV_73 | Clostridia | *Clostridium_sensu_stricto_1* | 0.545 | 1.087 | 1.423 | 0.435 | 0.492 | 0.719 | 1.253 | 2.210 | 1.977 | 0.000 | 0.000 | 0.000 | 0.000 | 0.000 | 0.000 |
| ASV_87 | Gammaproteobacteria | *Oligella* | 1.164 | -1.644 | -1.174 | 0.873 | 0.804 | 0.821 | 1.333 | -2.046 | -1.430 | 0.000 | 0.000 | 0.000 | 0.000 | 0.000 | 0.000 |
| ASV_91 | Negativicutes | *Megamonas* | -3.276 | -3.263 | -3.689 | 1.094 | 1.113 | 1.048 | -2.995 | -2.932 | -3.520 | 0.000 | 0.000 | 0.000 | 0.000 | 0.000 | 0.000 |
| ASV_98 | Bacteroidia | *Bacteroides* | -0.050 | 0.424 | -0.336 | 0.526 | 0.536 | 0.392 | -0.094 | 0.792 | -0.859 | 0.000 | 0.000 | 0.000 | 0.000 | 0.000 | 0.000 |
| ASV_107 | Actinobacteria | *Corynebacterium* | -0.618 | -2.392 | -1.518 | 0.889 | 0.814 | 0.879 | -0.694 | -2.937 | -1.727 | 0.000 | 0.000 | 0.000 | 0.000 | 0.000 | 0.000 |
| ASV_108 | Gammaproteobacteria | *Klebsiella* | 0.062 | -0.549 | -0.807 | 0.800 | 0.764 | 0.707 | 0.077 | -0.719 | -1.141 | 0.000 | 0.000 | 0.000 | 0.000 | 0.000 | 0.000 |
| ASV_110 | Bacteroidia | *Bacteroides* | 0.861 | 2.423 | 0.502 | 0.396 | 0.529 | 0.472 | 2.175 | 4.577 | 1.063 | 0.000 | 0.000 | 0.000 | 0.000 | 0.000 | 0.000 |
| ASV_113 | Bacteroidia | *NA* | 0.361 | -0.465 | -0.457 | 0.554 | 0.458 | 0.429 | 0.651 | -1.014 | -1.065 | 0.000 | 0.000 | 0.000 | 0.000 | 0.000 | 0.000 |
| ASV_118 | Clostridia | *Clostridium_sensu_stricto_1* | 0.462 | 0.496 | 2.623 | 0.425 | 0.380 | 0.895 | 1.088 | 1.305 | 2.931 | 0.000 | 0.000 | 0.000 | 0.000 | 0.000 | 0.000 |
| ASV_120 | Clostridia | *Helcococcus* | 0.869 | 1.676 | 0.019 | 0.414 | 0.536 | 0.191 | 2.101 | 3.128 | 0.100 | 0.000 | 0.000 | 0.000 | 0.000 | 0.000 | 0.000 |
| ASV_156 | Clostridia | *Peptoclostridium* | 0.109 | 1.154 | 0.306 | 0.268 | 0.455 | 0.302 | 0.406 | 2.535 | 1.012 | 0.000 | 0.000 | 0.000 | 0.000 | 0.000 | 0.000 |
| ASV_159 | Negativicutes | *Megasphaera* | -0.016 | 1.579 | 0.455 | 0.299 | 0.515 | 0.430 | -0.055 | 3.068 | 1.058 | 0.000 | 0.000 | 0.000 | 0.000 | 0.000 | 0.000 |
| ASV_164 | Clostridia | *Clostridium_sensu_stricto_1* | 0.242 | 0.502 | 1.200 | 0.370 | 0.349 | 0.627 | 0.655 | 1.441 | 1.915 | 0.000 | 0.000 | 0.000 | 0.000 | 0.000 | 0.000 |
| ASV_168 | Bacteroidia | *Bacteroides* | 0.465 | 0.776 | 0.430 | 0.377 | 0.380 | 0.299 | 1.233 | 2.045 | 1.438 | 0.000 | 0.000 | 0.000 | 0.000 | 0.000 | 0.000 |
| ASV_171 | Actinobacteria | *Dietzia* | -0.403 | -1.323 | -1.341 | 0.749 | 0.702 | 0.672 | -0.537 | -1.886 | -1.996 | 0.000 | 0.000 | 0.000 | 0.000 | 0.000 | 0.000 |
| ASV_174 | Clostridia | *Peptoclostridium* | 0.113 | 1.123 | 0.337 | 0.273 | 0.449 | 0.328 | 0.415 | 2.504 | 1.029 | 0.000 | 0.000 | 0.000 | 0.000 | 0.000 | 0.000 |
| ASV_177 | Actinobacteria | *Corynebacterium* | -2.760 | -3.082 | -3.047 | 0.977 | 0.964 | 0.940 | -2.824 | -3.196 | -3.241 | 0.000 | 0.000 | 0.000 | 0.000 | 0.000 | 0.000 |
| ASV_179 | Actinobacteria | *Corynebacterium* | 0.521 | -0.915 | 0.026 | 0.627 | 0.563 | 0.655 | 0.831 | -1.626 | 0.040 | 0.000 | 0.000 | 0.000 | 0.000 | 0.000 | 0.000 |
| ASV_186 | Bacteroidia | *Bacteroides* | 0.546 | 1.596 | 0.358 | 0.365 | 0.439 | 0.346 | 1.496 | 3.632 | 1.037 | 0.000 | 0.000 | 0.000 | 0.000 | 0.000 | 0.000 |
| ASV_190 | Clostridia | *Clostridium_sensu_stricto_7* | 0.042 | 0.791 | 0.166 | 0.322 | 0.406 | 0.234 | 0.130 | 1.947 | 0.706 | 0.000 | 0.000 | 0.000 | 0.000 | 0.000 | 0.000 |
| ASV_197 | Clostridia | *Fastidiosipila* | 0.593 | -1.357 | -0.513 | 0.722 | 0.666 | 0.800 | 0.820 | -2.038 | -0.641 | 0.000 | 0.000 | 0.000 | 0.000 | 0.000 | 0.000 |
| ASV_202 | Clostridia | *Hathewaya* | 0.089 | 0.821 | 0.381 | 0.280 | 0.451 | 0.411 | 0.317 | 1.820 | 0.928 | 0.000 | 0.000 | 0.000 | 0.000 | 0.000 | 0.000 |
| ASV_203 | Bacilli | *Ignavigranum* | -0.199 | -1.754 | -1.669 | 0.894 | 0.863 | 0.840 | -0.223 | -2.033 | -1.987 | 0.000 | 0.000 | 0.000 | 0.000 | 0.000 | 0.000 |
| ASV_207 | Actinobacteria | *Corynebacterium* | 0.665 | -0.552 | 0.389 | 0.583 | 0.487 | 0.581 | 1.141 | -1.135 | 0.669 | 0.000 | 0.000 | 0.000 | 0.000 | 0.000 | 0.000 |
| ASV_210 | Bacilli | *Enterococcus* | 1.142 | 0.089 | 0.755 | 0.401 | 0.283 | 0.494 | 2.849 | 0.314 | 1.528 | 0.000 | 0.000 | 0.000 | 0.000 | 0.000 | 0.000 |
| ASV_214 | Actinobacteria | *Corynebacterium* | 0.461 | -1.326 | -0.991 | 0.684 | 0.622 | 0.644 | 0.673 | -2.133 | -1.540 | 0.000 | 0.000 | 0.000 | 0.000 | 0.000 | 0.000 |
| ASV_216 | Clostridia | *Clostridium_sensu_stricto_7* | 0.149 | 0.446 | 0.660 | 0.335 | 0.344 | 0.539 | 0.446 | 1.295 | 1.225 | 0.000 | 0.000 | 0.000 | 0.000 | 0.000 | 0.000 |
| ASV_217 | Negativicutes | *Megamonas* | 0.508 | 0.483 | 0.436 | 0.384 | 0.373 | 0.442 | 1.322 | 1.294 | 0.986 | 0.000 | 0.000 | 0.000 | 0.000 | 0.000 | 0.000 |
| ASV_223 | Clostridia | *Fastidiosipila* | 0.742 | -0.066 | 0.714 | 0.339 | 0.206 | 0.539 | 2.190 | -0.321 | 1.324 | 0.000 | 0.000 | 0.000 | 0.000 | 0.000 | 0.000 |
| ASV_224 | Clostridia | *Lachnoclostridium* | 0.180 | 1.403 | 0.456 | 0.288 | 0.440 | 0.430 | 0.627 | 3.187 | 1.058 | 0.000 | 0.000 | 0.000 | 0.000 | 0.000 | 0.000 |
| ASV_235 | Clostridia | *Gallicola* | -0.765 | -1.316 | -1.123 | 0.759 | 0.737 | 0.721 | -1.008 | -1.785 | -1.557 | 0.000 | 0.000 | 0.000 | 0.000 | 0.000 | 0.000 |
| ASV_252 | Clostridia | *Peptostreptococcus* | -0.195 | 1.310 | 0.412 | 0.220 | 0.479 | 0.392 | -0.886 | 2.732 | 1.052 | 0.000 | 0.000 | 0.000 | 0.000 | 0.000 | 0.000 |
| ASV_269 | Clostridia | *Helcococcus* | 0.469 | -0.422 | -0.137 | 0.422 | 0.372 | 0.374 | 1.112 | -1.134 | -0.365 | 0.000 | 0.000 | 0.000 | 0.000 | 0.000 | 0.000 |
| ASV_274 | Clostridia | *NA* | 0.217 | 1.085 | 0.019 | 0.304 | 0.398 | 0.191 | 0.713 | 2.723 | 0.100 | 0.000 | 0.000 | 0.000 | 0.000 | 0.000 | 0.000 |
| ASV_277 | Bacteroidia | *Bacteroides* | 0.043 | 0.963 | 0.390 | 0.278 | 0.423 | 0.373 | 0.156 | 2.279 | 1.047 | 0.000 | 0.000 | 0.000 | 0.000 | 0.000 | 0.000 |
| ASV_283 | Actinobacteria | *Trueperella* | 0.675 | -0.380 | -0.030 | 0.424 | 0.337 | 0.410 | 1.591 | -1.127 | -0.072 | 0.000 | 0.000 | 0.000 | 0.000 | 0.000 | 0.000 |
| ASV_289 | Clostridia | *Lachnoclostridium* | 0.195 | 0.970 | 0.367 | 0.320 | 0.393 | 0.353 | 0.609 | 2.471 | 1.040 | 0.000 | 0.000 | 0.000 | 0.000 | 0.000 | 0.000 |
| ASV_306 | Gammaproteobacteria | *Acinetobacter* | 0.548 | 0.122 | 0.376 | 0.315 | 0.267 | 0.337 | 1.740 | 0.457 | 1.115 | 0.000 | 0.000 | 0.000 | 0.000 | 0.000 | 0.000 |
| ASV_323 | Gammaproteobacteria | *Proteus* | 0.663 | 0.966 | 0.019 | 0.348 | 0.390 | 0.191 | 1.905 | 2.480 | 0.100 | 0.000 | 0.000 | 0.000 | 0.000 | 0.000 | 0.000 |
| ASV_327 | Actinobacteria | *Flaviflexus* | -0.191 | -0.805 | -0.104 | 0.548 | 0.506 | 0.549 | -0.348 | -1.591 | -0.189 | 0.000 | 0.000 | 0.000 | 0.000 | 0.000 | 0.000 |
| ASV_341 | Actinobacteria | *Arthrobacter* | 0.287 | -0.508 | -0.284 | 0.475 | 0.420 | 0.421 | 0.605 | -1.211 | -0.675 | 0.000 | 0.000 | 0.000 | 0.000 | 0.000 | 0.000 |
| ASV_353 | Gammaproteobacteria | *Sutterella* | -0.206 | 0.404 | -0.175 | 0.303 | 0.413 | 0.264 | -0.679 | 0.980 | -0.666 | 0.000 | 0.000 | 0.000 | 0.000 | 0.000 | 0.000 |
| ASV_361 | Bacteroidia | *Parabacteroides* | -0.040 | 0.945 | 0.429 | 0.248 | 0.375 | 0.407 | -0.162 | 2.521 | 1.055 | 0.000 | 0.000 | 0.000 | 0.000 | 0.000 | 0.000 |
| ASV_363 | Bacilli | *Anoxybacillus* | 0.524 | 0.666 | 0.313 | 0.348 | 0.322 | 0.337 | 1.506 | 2.068 | 0.930 | 0.000 | 0.000 | 0.000 | 0.000 | 0.000 | 0.000 |
| ASV_393 | Actinobacteria | *Corynebacterium* | -0.250 | -1.385 | -1.114 | 0.677 | 0.638 | 0.635 | -0.370 | -2.171 | -1.754 | 0.000 | 0.000 | 0.000 | 0.000 | 0.000 | 0.000 |
| ASV_395 | Actinobacteria | *Leucobacter* | 0.179 | -0.744 | -0.659 | 0.489 | 0.437 | 0.416 | 0.365 | -1.704 | -1.585 | 0.000 | 0.000 | 0.000 | 0.000 | 0.000 | 0.000 |
| ASV_405 | Clostridia | *W5053* | 0.294 | -0.609 | 0.187 | 0.457 | 0.418 | 0.546 | 0.644 | -1.459 | 0.343 | 0.000 | 0.000 | 0.000 | 0.000 | 0.000 | 0.000 |
| ASV_408 | Clostridia | *Soehngenia* | 0.083 | -0.911 | -0.219 | 0.474 | 0.447 | 0.574 | 0.175 | -2.036 | -0.382 | 0.000 | 0.000 | 0.000 | 0.000 | 0.000 | 0.000 |
| ASV_444 | Clostridia | *Guggenheimella* | 0.181 | -0.403 | 0.450 | 0.388 | 0.356 | 0.540 | 0.467 | -1.131 | 0.832 | 0.000 | 0.000 | 0.000 | 0.000 | 0.000 | 0.000 |
| ASV_453 | Clostridia | *UBA1819* | -0.162 | 0.824 | 0.370 | 0.234 | 0.363 | 0.355 | -0.696 | 2.268 | 1.041 | 0.000 | 0.000 | 0.000 | 0.000 | 0.000 | 0.000 |
| ASV_474 | Clostridia | *Fastidiosipila* | 0.270 | -0.315 | 0.278 | 0.443 | 0.390 | 0.486 | 0.610 | -0.807 | 0.572 | 0.000 | 0.000 | 0.000 | 0.000 | 0.000 | 0.000 |
| ASV_527 | Gammaproteobacteria | *Halomonas* | 0.170 | 0.443 | 0.092 | 0.270 | 0.311 | 0.200 | 0.629 | 1.424 | 0.462 | 0.000 | 0.000 | 0.000 | 0.000 | 0.000 | 0.000 |
| ASV_553 | Actinobacteria | *Corynebacterium* | 0.394 | 0.441 | 0.179 | 0.304 | 0.274 | 0.263 | 1.298 | 1.611 | 0.682 | 0.000 | 0.000 | 0.000 | 0.000 | 0.000 | 0.000 |
| ASV_647 | Bacilli | *NA* | -0.714 | -0.931 | -0.327 | 0.573 | 0.552 | 0.598 | -1.246 | -1.686 | -0.547 | 0.000 | 0.000 | 0.000 | 0.000 | 0.000 | 0.000 |
| ASV_653 | Gammaproteobacteria | *Moraxella* | -0.059 | 0.585 | 0.239 | 0.234 | 0.293 | 0.259 | -0.252 | 1.998 | 0.922 | 0.000 | 0.000 | 0.000 | 0.000 | 0.000 | 0.000 |
| ASV_755 | Negativicutes | *Megasphaera* | -0.109 | 1.002 | 0.258 | 0.219 | 0.336 | 0.265 | -0.501 | 2.984 | 0.973 | 0.000 | 0.000 | 0.000 | 0.000 | 0.000 | 0.000 |
| ASV_915 | Actinobacteria | *Actinomyces* | -0.009 | -0.107 | -0.299 | 0.298 | 0.278 | 0.219 | -0.029 | -0.385 | -1.364 | 0.000 | 0.000 | 0.000 | 0.000 | 0.000 | 0.000 |
| ASV_1004 | Bacilli | *Staphylococcus* | -0.581 | -0.766 | -0.395 | 0.471 | 0.452 | 0.483 | -1.233 | -1.694 | -0.819 | 0.000 | 0.000 | 0.000 | 0.000 | 0.000 | 0.000 |

**Supplementary Table S8.** Summary of top linear regression models found using backwards model selection and Akaike information criterion (*AIC_c_*) scores. Models show significant terms (i.e. ecological factors) that explain variation in Shannon and Inverse Simpson alpha diversity, Faith’s phylogenetic diversity, and Bray-Curtis and weighted UniFrac beta diversity indices for the subset of Southern Beaufort Sea (SB) polar bears for which fatty acid diet data was available. There were no significant terms in the models predicting variation in weighted UniFrac beta diversity NMDS axes.

| **Shannon Alpha Diversity** (Adj. R^2^ = 0.16, *p* = 0.032) | | | | | |
| --- | --- | --- | --- | --- | --- |
| Top Model: | ~ Sex/age class* + Body Condition + FA_PC1* +FA_PC2 | | | | |
|  | *Coefficients* | *Estimate* | *Std.Error* | *tvalue* | *Pr(>\|t\|)* |
|  | (Intercept) | 2.37 | 3.3E-01 | 7.25 | 8.5E-09 |
|  | Sex/age class (Adult male) | -0.90 | 0.38 | -2.40 | **0.021** |
|  | Sex/age class (Subadult) | -0.34 | 0.21 | -1.62 | 0.11 |
|  | Body Condition | 0.003 | 0.0015 | 2.00 | **0.052** |
|  | FA_PC1 | -0.086 | 0.037 | -2.32 | **0.026** |
|  | FA_PC2 | 0.067 | 0.045 | 1.49 | 0.14 |
| **Inverse Simpson Alpha Diversity** (Adj. R^2^ = 0.10, *p* = 0.039) | | | | | |
| Top Model: | ~ FA_PC1* + FA_PC2 | | | | |
|  | *Coefficients* | *Estimate* | *Std.Error* | *tvalue* | *Pr(>\|t\|)* |
|  | (Intercept) | 0.86 | 0.011 | 78.80 | <2e-16 |
|  | FA_PC1 | -0.011 | 0.0048 | -2.19 | **0.034** |
|  | FA_PC2 | 0.011 | 0.0074 | 1.49 | 0.14 |
| **Faiths Phylogenetic Diversity** (Adj. R^2^ = 0.23, *p* = 0.003) | | | | | |
| Top Model: | ~ Sex/age class * + FA_PC1 | | | | |
|  | *Coefficients* | *Estimate* | *Std.Error* | *tvalue* | *Pr(>\|t\|)* |
|  | (Intercept) | 15.66 | 0.66 | 23.78 | < |
|  | Sex/age class (Adult male) | -3.26 | 0.95 | -3.44 | **0.001** |
|  | Sex/age class (Subadult) | -3.53 | 1.12 | -3.15 | **0.003** |
|  | FA_PC1 | -0.28 | 0.20 | -1.43 | 0.16 |
| **Beta Diversity: Bray-Curtis NMDS 1** (Adj. R2 = 0.15, p = 0.011) | | | | | |
|  | | | | | |
| Top Model: | ~ Sex/age class * + FA_PC1 | | | | |
|  | *Coefficients* | *Estimate* | *Std.Error* | *tvalue* | *Pr(>\|t\|)* |
|  | (Intercept) | 0.16 | 0.065 | 2.42 | 0.020 |
|  | Sex/age class (Adult male) | -0.22 | 0.084 | -2.58 | **0.013** |
|  | Sex/age class (Subadult) | -0.34 | 0.12 | -2.74 | **0.009** |
| **Beta Diversity: Bray-Curtis NMDS 2** (Adj. R2 = 0.14, p = 0.014) | | | | | |
| Top Model: | ~ FA_PC2 **.** + Capture year* | | | | |
|  | *Coefficients* | *Estimate* | *Std.Error* | *tvalue* | *Pr(>\|t\|)* |
|  | (Intercept) | 124.0 | 58.35 | 2.13 | 0.039 |
|  | FA_PC2 | -0.040 | 0.022 | -1.84 | **0.072** |
|  | Capture year | -0.061 | 0.029 | -2.13 | **0.039** |
| **Beta Diversity: Weighted UniFrac NMDS1** (Adj. R2 = -0.029, p = 0.58) | | | | | |
| Full Model: | ~ Sex/age class + Condition + FA_PC1 + FA_PC2 + Capture year | | | | |
|  | *Coefficients* | *Estimate* | *Std.Error* | *tvalue* | *Pr(>\|t\|)* |
|  | (Intercept) | 1.8 | 0.10 | 0.18 | 0.86 |
|  | Sex/age class (Adult male) | 1.1E-02 | 2.8E-02 | 0.41 | 0.69 |
|  | Sex/age class (Subadult) | 2.9E-03 | 1.5E-02 | 0.19 | 0.85 |
|  | Body Condition | -1.9E-05 | 1.1E-04 | -0.17 | 0.86 |
|  | FA_PC1 | 3.9E-03 | 3.0E-03 | 1.29 | 0.20 |
|  | FA_PC2 | -4.2E-03 | 3.3E-03 | -1.29 | 0.20 |
|  | Capture year | -9.2E-04 | 5.1E-03 | -0.18 | 0.86 |
| **Beta Diversity: Weighted UniFrac NMDS2** (Adj. R2 = -0.069, p = 0.79) | | | | | |
| Full Model: | ~ Sex/age class + Body Condition + FA_PC1 + FA_PC2 + Capture year | | | | |
|  | *Coefficients* | *Estimate* | *Std.Error* | *tvalue* | *Pr(>\|t\|)* |
|  | (Intercept) | 5.4E+00 | 6.99E+00 | 0.777 | 0.44 |
|  | Sex/age class (Adult male) | 1.4E-04 | 1.9E-02 | 0.007 | 0.99 |
|  | Sex/age class (Subadult) | 1.5E-02 | 1.0E-02 | 1.42 | 0.16 |
|  | Body Condition | 9.3E-06 | 7.7E-05 | 0.12 | 0.90 |
|  | FA_PC1 | 3.3E-05 | 2.1E-03 | 0.016 | 0.99 |
|  | FA_PC2 | 1.2E-03 | 2.2E-03 | 0.54 | 0.60 |
|  | Capture year | -2.7E-03 | 3.5E-03 | -0.78 | 0.44 |

**Supplementary Table S9.** Results of Analysis of Composition with bias correction (ANCOM-BC) analysis for the 25 bacterial genera that significantly differed in abundance among polar bear sex/age classes sex/age classes (adult males [AM] and subadults [S] compared to adult females [AF], for East Greenland (EG) and Southern Beaufort Sea (SB) polar bear subpopulations (False discovery rate (FDR) adjusted *p*-value cutoff: 0.05)

|  | Genus | Coef.  AM-AF | Coef.  S - AF | SE  AM - AF | SE  S - AF | W  Statistic  AM - AF | W  Statistic  S - AF | *p* -value  AM - AF | *p -*value  S - AF | FDR  Adj.  *p* -value  AM - AF | FDR  Adj.  *p* -value  S - AF |
| --- | --- | --- | --- | --- | --- | --- | --- | --- | --- | --- | --- |
| 1 | *Brachybacterium* | -1.402 | -0.984 | 0.589 | 0.603 | -2.381 | -1.631 | 0.000 | 0.000 | 0.000 | 0.000 |
| 2 | *Helcobacillus* | -2.347 | -2.146 | 0.651 | 0.642 | -3.608 | -3.344 | 0.000 | 0.000 | 0.000 | 0.000 |
| 3 | *Arthrobacter* | -1.062 | 0.613 | 0.468 | 1.201 | -2.270 | 0.510 | 0.000 | 0.000 | 0.000 | 0.000 |
| 4 | *Atopobium* | 0.666 | 1.704 | 0.360 | 0.972 | 1.851 | 1.753 | 0.000 | 0.000 | 0.000 | 0.000 |
| 5 | *Proteiniphilum* | -0.371 | 0.013 | 0.410 | 0.379 | -0.905 | 0.035 | 0.000 | 0.000 | 0.000 | 0.000 |
| 6 | *Chryseobacterium* | -1.093 | -0.592 | 0.574 | 0.589 | -1.904 | -1.005 | 0.000 | 0.000 | 0.000 | 0.000 |
| 7 | *Bacillus* | -0.286 | -0.069 | 0.462 | 0.410 | -0.619 | -0.168 | 0.000 | 0.000 | 0.000 | 0.000 |
| 8 | *Ignavigranum* | -2.585 | -1.976 | 0.533 | 0.561 | -4.850 | -3.523 | 0.000 | 0.000 | 0.000 | 0.000 |
| 9 | *Vagococcus* | -1.673 | -1.064 | 0.764 | 0.784 | -2.191 | -1.358 | 0.000 | 0.000 | 0.000 | 0.000 |
| 10 | *Nosocomiicoccus* | -3.914 | -2.793 | 0.638 | 0.919 | -6.130 | -3.040 | 0.000 | 0.002 | 0.000 | 0.241 |
| 11 | *Hathewaya* | 0.515 | 0.126 | 0.606 | 0.367 | 0.849 | 0.345 | 0.000 | 0.000 | 0.000 | 0.000 |
| 12 | *Epulopiscium* | -0.949 | 0.203 | 0.568 | 0.754 | -1.670 | 0.269 | 0.000 | 0.000 | 0.000 | 0.000 |
| 13 | *Colidextribacter* | 0.894 | 1.056 | 0.392 | 0.342 | 2.282 | 3.091 | 0.000 | 0.000 | 0.000 | 0.000 |
| 14 | *UCG_005* | 0.355 | 0.492 | 0.400 | 0.388 | 0.886 | 1.266 | 0.000 | 0.000 | 0.000 | 0.000 |
| 15 | *Gallicola* | -1.005 | -0.396 | 0.440 | 0.474 | -2.282 | -0.836 | 0.000 | 0.000 | 0.000 | 0.000 |
| 16 | *Soehngenia* | -1.249 | -0.640 | 0.387 | 0.424 | -3.230 | -1.508 | 0.000 | 0.000 | 0.000 | 0.000 |
| 17 | *W5053* | -1.442 | -0.833 | 0.405 | 0.441 | -3.565 | -1.891 | 0.000 | 0.000 | 0.000 | 0.000 |
| 18 | *Phascolarctobacterium* | 1.203 | 1.036 | 0.510 | 0.329 | 2.358 | 3.148 | 0.000 | 0.000 | 0.000 | 0.000 |
| 19 | *Oligella* | -3.116 | -2.025 | 0.646 | 0.765 | -4.823 | -2.649 | 0.000 | 0.008 | 0.000 | 0.799 |
| 20 | *Burkholderia_Caballeronia Paraburkholderia* | 0.391 | 0.473 | 0.362 | 0.326 | 1.077 | 1.450 | 0.000 | 0.000 | 0.000 | 0.000 |
| 21 | *Ottowia* | -0.050 | 0.154 | 0.452 | 0.456 | -0.111 | 0.337 | 0.000 | 0.000 | 0.000 | 0.000 |
| 22 | *Schlegelella* | -0.174 | 0.173 | 0.313 | 0.347 | -0.554 | 0.497 | 0.000 | 0.000 | 0.000 | 0.000 |
| 23 | *Cronobacter* | -0.347 | -0.001 | 0.515 | 0.517 | -0.674 | -0.002 | 0.000 | 0.000 | 0.000 | 0.000 |
| 24 | *Edwardsiella* | 0.444 | -0.307 | 0.748 | 0.552 | 0.593 | -0.556 | 0.000 | 0.000 | 0.000 | 0.000 |
| 25 | *Stenotrophomonas* | 0.249 | 0.317 | 0.414 | 0.344 | 0.602 | 0.920 | 0.000 | 0.000 | 0.000 | 0.000 |

**Supplementary Table S10.** Results of Analysis of Composition with bias correction (ANCOM-BC) analysis for the 99 amplicon sequence variants (ASVs) that significantly differed in abundance among polar bear sex/age classes (adult males [AM] and subadults [S] compared to adult females [AF], for East Greenland (EG) and Southern Beaufort Sea (SB) polar bear subpopulations (False discovery rate (FDR) adjusted *p*-value cutoff: 0.05)

|  | ASV | Class | Genus | Coef.  AM-AF | Coef.  S - AF | SE  AM - AF | SE  S - AF | W  Statistic  AM - AF | W  Statistic  S - AF | *p* -value  AM - AF | *p -*value  S - AF | FDR  Adj.  *p* -value  AM - AF | FDR  Adj.  *p* -value  S - AF |
| --- | --- | --- | --- | --- | --- | --- | --- | --- | --- | --- | --- | --- | --- |
| 1 | ASV_22 | Clostridia | *Fastidiosipila* | -4.34 | -3.40 | 0.76 | 1.48 | -5.70 | -2.30 | 0.00 | 0.02 | 0.00 | 1.00 |
| 2 | ASV_35 | Clostridia | *Clostridium_sensu_stricto_1* | 0.11 | -1.29 | 0.88 | 0.65 | 0.12 | -1.98 | 0.00 | 0.00 | 0.00 | 0.00 |
| 3 | ASV_40 | Gammaproteobacteria | *Edwardsiella* | 0.30 | -0.17 | 0.61 | 0.42 | 0.50 | -0.39 | 0.00 | 0.00 | 0.00 | 0.00 |
| 4 | ASV_42 | Gammaproteobacteria | *Psychrobacter* | -3.29 | -3.28 | 0.81 | 0.79 | -4.06 | -4.14 | 0.00 | 0.00 | 0.00 | 0.00 |
| 5 | ASV_43 | Clostridia | *Murdochiella* | 0.79 | -1.79 | 1.08 | 0.86 | 0.73 | -2.09 | 0.00 | 0.00 | 0.00 | 0.00 |
| 6 | ASV_60 | Clostridia | *Clostridium_sensu_stricto_1* | -0.85 | -1.45 | 0.69 | 0.60 | -1.23 | -2.43 | 0.00 | 0.00 | 0.00 | 0.00 |
| 7 | ASV_76 | Gammaproteobacteria | *Edwardsiella* | 0.29 | -0.21 | 0.64 | 0.49 | 0.46 | -0.43 | 0.00 | 0.00 | 0.00 | 0.00 |
| 8 | ASV_83 | Bacilli | *Nosocomiicoccus* | -3.67 | -2.79 | 0.64 | 0.89 | -5.70 | -3.11 | 0.00 | 0.00 | 0.00 | 0.50 |
| 9 | ASV_87 | Gammaproteobacteria | *Oligella* | -3.02 | -2.65 | 0.63 | 0.65 | -4.83 | -4.08 | 0.00 | 0.00 | 0.00 | 0.00 |
| 10 | ASV_91 | Negativicutes | *Megamonas* | -0.10 | -0.44 | 0.69 | 0.59 | -0.14 | -0.76 | 0.00 | 0.00 | 0.00 | 0.00 |
| 11 | ASV_96 | Actinobacteria | *Brevibacterium* | -3.04 | -2.20 | 0.59 | 0.86 | -5.15 | -2.55 | 0.00 | 0.01 | 0.00 | 1.00 |
| 12 | ASV_98 | Bacteroidia | *Bacteroides* | 0.34 | -0.47 | 0.70 | 0.57 | 0.49 | -0.83 | 0.00 | 0.00 | 0.00 | 0.00 |
| 13 | ASV_100 | Actinobacteria | *Helcobacillus* | -1.19 | -1.04 | 0.58 | 0.59 | -2.05 | -1.78 | 0.00 | 0.00 | 0.00 | 0.00 |
| 14 | ASV_103 | Bacilli | *Vagococcus* | -1.76 | -1.39 | 0.73 | 0.75 | -2.40 | -1.84 | 0.00 | 0.00 | 0.00 | 0.00 |
| 15 | ASV_104 | Actinobacteria | *Brevibacterium* | -2.30 | -2.26 | 0.67 | 0.66 | -3.46 | -3.42 | 0.00 | 0.00 | 0.00 | 0.00 |
| 16 | ASV_105 | Clostridia | *Fastidiosipila* | -0.02 | -2.51 | 0.87 | 0.69 | -0.03 | -3.64 | 0.00 | 0.00 | 0.00 | 0.00 |
| 17 | ASV_107 | Actinobacteria | *Corynebacterium* | -1.82 | -1.45 | 0.64 | 0.67 | -2.83 | -2.18 | 0.00 | 0.00 | 0.00 | 0.00 |
| 18 | ASV_108 | Gammaproteobacteria | *Klebsiella* | -0.24 | -0.35 | 0.54 | 0.48 | -0.44 | -0.73 | 0.00 | 0.00 | 0.00 | 0.00 |
| 19 | ASV_113 | Bacteroidia | *NA* | -1.11 | -0.85 | 0.60 | 0.62 | -1.85 | -1.38 | 0.00 | 0.00 | 0.00 | 0.00 |
| 20 | ASV_114 | Actinobacteria | *Helcobacillus* | -2.08 | -1.90 | 0.61 | 0.61 | -3.43 | -3.08 | 0.00 | 0.00 | 0.00 | 0.00 |
| 21 | ASV_119 | Actinobacteria | *Flaviflexus* | -0.36 | -1.54 | 0.82 | 0.69 | -0.45 | -2.24 | 0.00 | 0.00 | 0.00 | 0.00 |
| 22 | ASV_120 | Clostridia | *Helcococcus* | -0.09 | -1.97 | 0.91 | 0.71 | -0.10 | -2.77 | 0.00 | 0.00 | 0.00 | 0.00 |
| 23 | ASV_123 | Fusobacteriia | *Fusobacterium* | -0.71 | -1.03 | 0.71 | 0.61 | -1.00 | -1.68 | 0.00 | 0.00 | 0.00 | 0.00 |
| 24 | ASV_128 | Fusobacteriia | *Fusobacterium* | -0.06 | -0.43 | 0.58 | 0.46 | -0.10 | -0.93 | 0.00 | 0.00 | 0.00 | 0.00 |
| 25 | ASV_139 | Gammaproteobacteria | *Klebsiella* | 0.34 | -0.16 | 0.59 | 0.45 | 0.58 | -0.36 | 0.00 | 0.00 | 0.00 | 0.00 |
| 26 | ASV_142 | Gammaproteobacteria | *Acinetobacter* | 0.31 | 0.17 | 0.32 | 0.28 | 0.95 | 0.60 | 0.00 | 0.00 | 0.00 | 0.00 |
| 27 | ASV_143 | Actinobacteria | *Leucobacter* | -1.91 | -1.81 | 0.66 | 0.67 | -2.91 | -2.72 | 0.00 | 0.00 | 0.00 | 0.00 |
| 28 | ASV_148 | Actinobacteria | *NA* | -2.18 | -1.44 | 0.58 | 0.66 | -3.79 | -2.16 | 0.00 | 0.03 | 0.04 | 1.00 |
| 29 | ASV_152 | Clostridia | *Romboutsia* | 0.27 | -0.28 | 0.56 | 0.46 | 0.49 | -0.61 | 0.00 | 0.00 | 0.00 | 0.00 |
| 30 | ASV_157 | Actinobacteria | *Brachybacterium* | -1.49 | -1.31 | 0.58 | 0.59 | -2.57 | -2.21 | 0.00 | 0.00 | 0.00 | 0.00 |
| 31 | ASV_170 | Saccharimonadia | *NA* | -0.46 | -0.19 | 0.35 | 0.37 | -1.32 | -0.52 | 0.00 | 0.00 | 0.00 | 0.00 |
| 32 | ASV_179 | Actinobacteria | *Corynebacterium* | -2.47 | -1.72 | 0.52 | 0.69 | -4.74 | -2.47 | 0.00 | 0.00 | 0.00 | 0.00 |
| 33 | ASV_184 | Bacteroidia | *Bacteroides* | -0.85 | -0.78 | 0.60 | 0.59 | -1.40 | -1.32 | 0.00 | 0.00 | 0.00 | 0.00 |
| 34 | ASV_189 | Clostridia | *Peptoniphilus* | -2.09 | -1.17 | 0.53 | 0.81 | -3.93 | -1.43 | 0.00 | 0.15 | 0.02 | 1.00 |
| 35 | ASV_197 | Clostridia | *Fastidiosipila* | -2.85 | -1.63 | 0.52 | 0.94 | -5.49 | -1.73 | 0.00 | 0.00 | 0.00 | 0.00 |
| 36 | ASV_199 | Bacteroidia | *Bacteroides* | -1.25 | -1.09 | 0.65 | 0.64 | -1.91 | -1.69 | 0.00 | 0.00 | 0.00 | 0.00 |
| 37 | ASV_202 | Clostridia | *Hathewaya* | 0.43 | -0.20 | 0.59 | 0.35 | 0.73 | -0.56 | 0.00 | 0.00 | 0.00 | 0.00 |
| 38 | ASV_203 | Bacilli | *Ignavigranum* | -2.65 | -2.28 | 0.51 | 0.54 | -5.23 | -4.25 | 0.00 | 0.00 | 0.00 | 0.00 |
| 39 | ASV_207 | Actinobacteria | *Corynebacterium* | -2.40 | -1.69 | 0.55 | 0.65 | -4.36 | -2.60 | 0.00 | 0.00 | 0.00 | 0.00 |
| 40 | ASV_209 | Actinobacteria | *Corynebacterium* | 0.71 | 0.04 | 0.57 | 0.40 | 1.25 | 0.09 | 0.00 | 0.00 | 0.00 | 0.00 |
| 41 | ASV_214 | Actinobacteria | *Corynebacterium* | -2.18 | -1.81 | 0.56 | 0.58 | -3.90 | -3.09 | 0.00 | 0.00 | 0.00 | 0.00 |
| 42 | ASV_223 | Clostridia | *Fastidiosipila* | -1.27 | -0.47 | 0.47 | 0.62 | -2.72 | -0.76 | 0.00 | 0.00 | 0.00 | 0.00 |
| 43 | ASV_230 | Clostridia | *Peptoclostridium* | 0.72 | 0.03 | 0.53 | 0.39 | 1.35 | 0.07 | 0.00 | 0.00 | 0.00 | 0.00 |
| 44 | ASV_235 | Clostridia | *Gallicola* | -0.91 | -0.55 | 0.41 | 0.44 | -2.26 | -1.24 | 0.00 | 0.00 | 0.00 | 0.00 |
| 45 | ASV_237 | Bacteroidia | *Porphyromonas* | 0.21 | -0.10 | 0.57 | 0.54 | 0.37 | -0.18 | 0.00 | 0.00 | 0.00 | 0.00 |
| 46 | ASV_240 | Clostridia | *NA* | 1.61 | 0.37 | 0.55 | 0.26 | 2.95 | 1.43 | 0.00 | 0.00 | 0.00 | 0.00 |
| 47 | ASV_244 | Bacilli | *Facklamia* | -0.74 | -0.78 | 0.62 | 0.57 | -1.19 | -1.38 | 0.00 | 0.00 | 0.00 | 0.00 |
| 48 | ASV_249 | Bacteroidia | *Porphyromonas* | -1.53 | -1.49 | 0.56 | 0.54 | -2.72 | -2.76 | 0.00 | 0.00 | 0.00 | 0.00 |
| 49 | ASV_251 | Clostridia | *Murdochiella* | 0.67 | 0.09 | 0.56 | 0.39 | 1.20 | 0.24 | 0.00 | 0.00 | 0.00 | 0.00 |
| 50 | ASV_253 | Clostridia | *Clostridium_sensu_stricto_1* | -0.38 | -0.32 | 0.52 | 0.49 | -0.73 | -0.65 | 0.00 | 0.00 | 0.00 | 0.00 |
| 51 | ASV_262 | Bacteroidia | *Bacteroides* | -1.03 | -0.94 | 0.66 | 0.63 | -1.56 | -1.50 | 0.00 | 0.00 | 0.00 | 0.00 |
| 52 | ASV_269 | Clostridia | *Helcococcus* | -1.27 | -0.90 | 0.41 | 0.44 | -3.12 | -2.04 | 0.00 | 0.00 | 0.00 | 0.00 |
| 53 | ASV_274 | Clostridia | *NA* | 0.41 | -0.74 | 0.66 | 0.55 | 0.62 | -1.35 | 0.00 | 0.00 | 0.00 | 0.00 |
| 54 | ASV_283 | Actinobacteria | *Trueperella* | -1.80 | -1.43 | 0.47 | 0.50 | -3.83 | -2.86 | 0.00 | 0.00 | 0.00 | 0.00 |
| 55 | ASV_303 | Clostridia | *Tuzzerella* | -0.19 | -0.05 | 0.34 | 0.35 | -0.54 | -0.15 | 0.00 | 0.00 | 0.00 | 0.00 |
| 56 | ASV_307 | Clostridia | *Murdochiella* | -0.24 | -0.61 | 0.67 | 0.58 | -0.35 | -1.05 | 0.00 | 0.00 | 0.00 | 0.00 |
| 57 | ASV_318 | Bacilli | *Streptococcus* | 0.43 | -0.15 | 0.51 | 0.42 | 0.83 | -0.37 | 0.00 | 0.00 | 0.00 | 0.00 |
| 58 | ASV_323 | Gammaproteobacteria | *Proteus* | -0.44 | -0.91 | 0.60 | 0.52 | -0.73 | -1.75 | 0.00 | 0.00 | 0.00 | 0.00 |
| 59 | ASV_327 | Actinobacteria | *Flaviflexus* | -1.19 | -0.26 | 0.46 | 0.60 | -2.59 | -0.44 | 0.00 | 0.00 | 0.00 | 0.00 |
| 60 | ASV_330 | Clostridia | *Peptoclostridium* | 0.72 | 0.02 | 0.54 | 0.40 | 1.32 | 0.04 | 0.00 | 0.00 | 0.00 | 0.00 |
| 61 | ASV_341 | Actinobacteria | *Arthrobacter* | -1.09 | -0.72 | 0.44 | 0.47 | -2.48 | -1.53 | 0.00 | 0.00 | 0.00 | 0.00 |
| 62 | ASV_343 | Clostridia | *Anaerococcus* | 0.20 | -0.25 | 0.58 | 0.49 | 0.34 | -0.52 | 0.00 | 0.00 | 0.00 | 0.00 |
| 63 | ASV_345 | Bacilli | *Nosocomiicoccus* | -1.45 | -1.08 | 0.57 | 0.60 | -2.52 | -1.80 | 0.00 | 0.00 | 0.00 | 0.00 |
| 64 | ASV_353 | Gammaproteobacteria | *Sutterella* | 0.14 | -0.12 | 0.50 | 0.41 | 0.27 | -0.29 | 0.00 | 0.00 | 0.00 | 0.00 |
| 65 | ASV_369 | Desulfovibrionia | *Bilophila* | 0.96 | 1.20 | 0.38 | 0.67 | 2.55 | 1.80 | 0.00 | 0.00 | 0.00 | 0.00 |
| 66 | ASV_393 | Actinobacteria | *Corynebacterium* | -1.65 | -1.28 | 0.49 | 0.52 | -3.40 | -2.48 | 0.00 | 0.00 | 0.00 | 0.00 |
| 67 | ASV_395 | Actinobacteria | *Leucobacter* | -1.02 | -0.65 | 0.39 | 0.43 | -2.64 | -1.54 | 0.00 | 0.00 | 0.00 | 0.00 |
| 68 | ASV_397 | Gammaproteobacteria | *Psychrobacter* | -0.37 | -0.28 | 0.43 | 0.40 | -0.84 | -0.70 | 0.00 | 0.00 | 0.00 | 0.00 |
| 69 | ASV_401 | Gammaproteobacteria | *Oligella* | -1.26 | -0.89 | 0.45 | 0.48 | -2.79 | -1.84 | 0.00 | 0.00 | 0.00 | 0.00 |
| 70 | ASV_405 | Clostridia | *W5053* | -1.53 | -1.16 | 0.39 | 0.43 | -3.89 | -2.69 | 0.00 | 0.00 | 0.00 | 0.00 |
| 71 | ASV_408 | Clostridia | *Soehngenia* | -1.33 | -0.96 | 0.37 | 0.41 | -3.62 | -2.36 | 0.00 | 0.00 | 0.00 | 0.00 |
| 72 | ASV_409 | Clostridia | *Peptoclostridium* | 0.07 | -0.22 | 0.54 | 0.46 | 0.12 | -0.48 | 0.00 | 0.00 | 0.00 | 0.00 |
| 73 | ASV_410 | Clostridia | *Helcococcus* | -1.10 | -0.30 | 0.39 | 0.57 | -2.83 | -0.53 | 0.00 | 0.00 | 0.00 | 0.00 |
| 74 | ASV_422 | Gracilibacteria | *NA* | -0.88 | -0.99 | 0.51 | 0.50 | -1.73 | -1.97 | 0.00 | 0.00 | 0.00 | 0.00 |
| 75 | ASV_474 | Clostridia | *Fastidiosipila* | -0.91 | -0.16 | 0.40 | 0.46 | -2.28 | -0.35 | 0.00 | 0.00 | 0.00 | 0.00 |
| 76 | ASV_502 | Actinobacteria | *NA* | 0.48 | 0.14 | 0.43 | 0.36 | 1.10 | 0.38 | 0.00 | 0.00 | 0.00 | 0.00 |
| 77 | ASV_511 | Clostridia | *Blautia* | -0.25 | -0.26 | 0.42 | 0.39 | -0.60 | -0.67 | 0.00 | 0.00 | 0.00 | 0.00 |
| 78 | ASV_516 | Bacilli | *Staphylococcus* | 0.27 | -0.01 | 0.44 | 0.37 | 0.61 | -0.02 | 0.00 | 0.00 | 0.00 | 0.00 |
| 79 | ASV_538 | Negativicutes | *Megasphaera* | 1.10 | 1.17 | 0.43 | 0.64 | 2.54 | 1.83 | 0.00 | 0.00 | 0.00 | 0.00 |
| 80 | ASV_547 | Bacilli | *Streptococcus* | -0.40 | -0.45 | 0.55 | 0.47 | -0.73 | -0.96 | 0.00 | 0.00 | 0.00 | 0.00 |
| 81 | ASV_551 | Coriobacteriia | *Atopobium* | 0.52 | 0.78 | 0.33 | 0.45 | 1.60 | 1.75 | 0.00 | 0.00 | 0.00 | 0.00 |
| 82 | ASV_563 | Clostridia | *NA* | 0.49 | 0.14 | 0.45 | 0.35 | 1.09 | 0.41 | 0.00 | 0.00 | 0.00 | 0.00 |
| 83 | ASV_587 | Actinobacteria | *Corynebacterium* | 0.44 | 0.15 | 0.45 | 0.37 | 0.99 | 0.41 | 0.00 | 0.00 | 0.00 | 0.00 |
| 84 | ASV_612 | Clostridia | *Epulopiscium* | -1.03 | -0.12 | 0.52 | 0.72 | -1.99 | -0.17 | 0.00 | 0.00 | 0.00 | 0.00 |
| 85 | ASV_646 | Gammaproteobacteria | *Schlegelella* | -0.26 | -0.15 | 0.29 | 0.32 | -0.88 | -0.47 | 0.00 | 0.00 | 0.00 | 0.00 |
| 86 | ASV_721 | Bacteroidia | *Chryseobacterium* | -0.78 | -0.41 | 0.34 | 0.38 | -2.32 | -1.08 | 0.00 | 0.00 | 0.00 | 0.00 |
| 87 | ASV_758 | Gammaproteobacteria | *Ottowia* | -0.13 | -0.17 | 0.43 | 0.44 | -0.31 | -0.39 | 0.00 | 0.00 | 0.00 | 0.00 |
| 88 | ASV_776 | Actinobacteria | *Corynebacterium* | 0.16 | -0.34 | 0.41 | 0.37 | 0.39 | -0.93 | 0.00 | 0.00 | 0.00 | 0.00 |
| 89 | ASV_810 | Alphaproteobacteria | *Methylobacterium-Methylorubrum* | 0.35 | 0.26 | 0.35 | 0.29 | 1.01 | 0.91 | 0.00 | 0.00 | 0.00 | 0.00 |
| 90 | ASV_833 | Gammaproteobacteria | *Burkholderia-Caballeronia-Paraburkholderia* | 0.31 | 0.15 | 0.32 | 0.29 | 0.95 | 0.51 | 0.00 | 0.00 | 0.00 | 0.00 |
| 91 | ASV_897 | Negativicutes | *Megamonas* | 0.25 | 0.15 | 0.35 | 0.32 | 0.71 | 0.46 | 0.00 | 0.00 | 0.00 | 0.00 |
| 92 | ASV_915 | Actinobacteria | *Actinomyces* | -0.27 | -0.18 | 0.37 | 0.38 | -0.73 | -0.46 | 0.00 | 0.00 | 0.00 | 0.00 |
| 93 | ASV_936 | Gammaproteobacteria | *Enterobacter* | -0.34 | -0.19 | 0.39 | 0.39 | -0.86 | -0.49 | 0.00 | 0.00 | 0.00 | 0.00 |
| 94 | ASV_1027 | Actinobacteria | *Dietzia* | 0.50 | 0.73 | 0.31 | 0.41 | 1.63 | 1.78 | 0.00 | 0.00 | 0.00 | 0.00 |
| 95 | ASV_1160 | Clostridia | *NA* | 0.64 | 0.92 | 0.29 | 0.42 | 2.18 | 2.17 | 0.00 | 0.00 | 0.00 | 0.00 |
| 96 | ASV_1321 | Clostridia | *Colidextribacter* | 0.52 | 0.73 | 0.31 | 0.28 | 1.67 | 2.61 | 0.00 | 0.00 | 0.00 | 0.00 |
| 97 | ASV_1541 | Bacteroidia | *Bacteroides* | -0.01 | 0.04 | 0.36 | 0.35 | -0.03 | 0.13 | 0.00 | 0.00 | 0.00 | 0.00 |
| 98 | ASV_1850 | Bacteroidia | *Parabacteroides* | -0.21 | -0.09 | 0.33 | 0.35 | -0.66 | -0.27 | 0.00 | 0.00 | 0.00 | 0.00 |
| 99 | ASV_1941 | Negativicutes | *Megasphaera* | 0.28 | 0.27 | 0.27 | 0.28 | 1.03 | 0.96 | 0.00 | 0.00 | 0.00 | 0.00 |

B)

A)


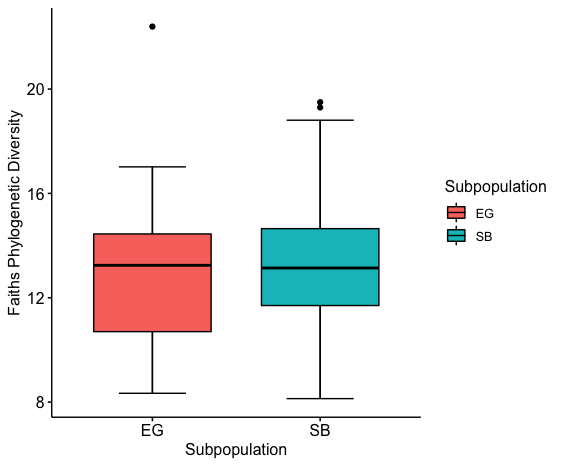

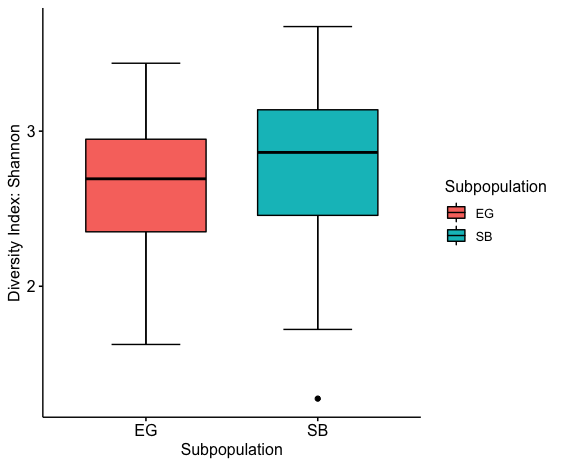


**Supplementary Figure S1.** Boxplots showing distribution of alpha diversity indices for East Greenland (EG) and Southern Beaufort Sea (SB) polar bears for (A) Shannon alpha diversity indices (Group means: EG: 2.65 ± 0.07; SB: 2.74 ± 0.06), and (B) Faith’s Phylogenetic Diversity indices (Group means: EG: 12.9 ± 0.5; SB: 13.3 ± 0.4).

B)

A)


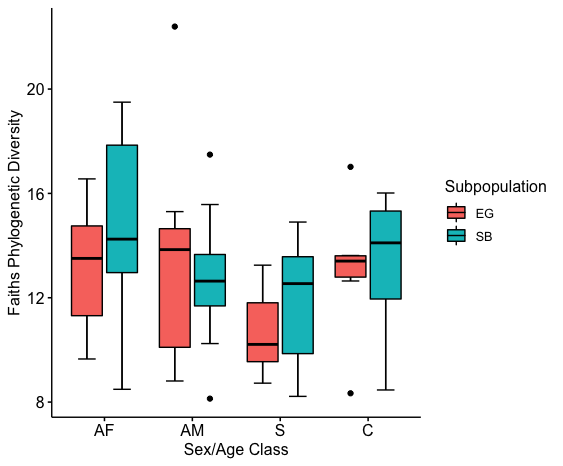

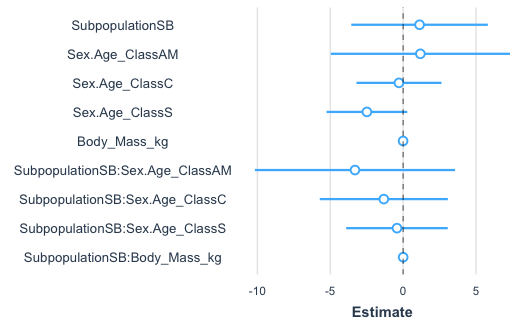


**Supplementary Figure S2.** (A) Boxplots showing Faith’s phylogenetic diversity (FPD) indices among sex/age classes (Adult females [AF], adult males [AM], subadults [S], and cubs [C]) for East Greenland (EG) (Group means: AF: 13.2 ± 0.5, AM: 13.5 ± 1.8, S: 10.7 ± 0.8, C: 13.1 ± 1.1) and Southern Beaufort Sea (SB) (Group means: AF: 14.8 ± 0.7, AM: 12.7 ± 0.4, S: 11.9 ± 0.7, C: 13.2 ± 1.7) polar bears. (B) Horizontal barplots summarizing linear regression coefficients for FPD regression model. Bars indicate magnitudes of variable effects and 95% confidence intervals for terms included in the FPD linear regression model.


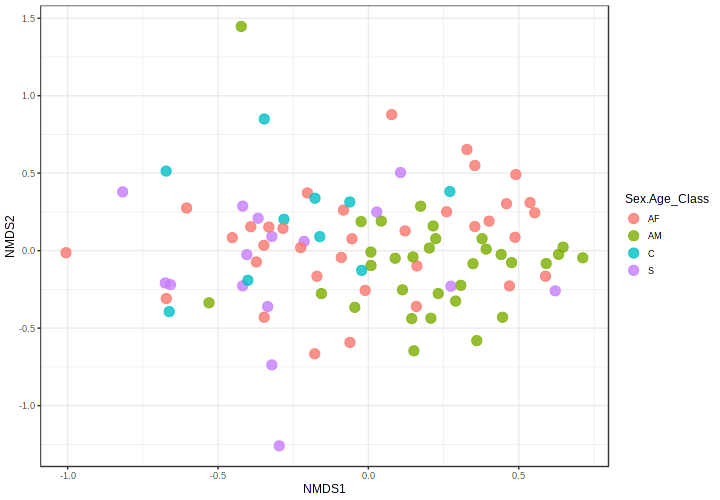


-1.0

-0.5

0.0

0.5

1.0

1.5

-1.0

-0.5

0.0

0.5


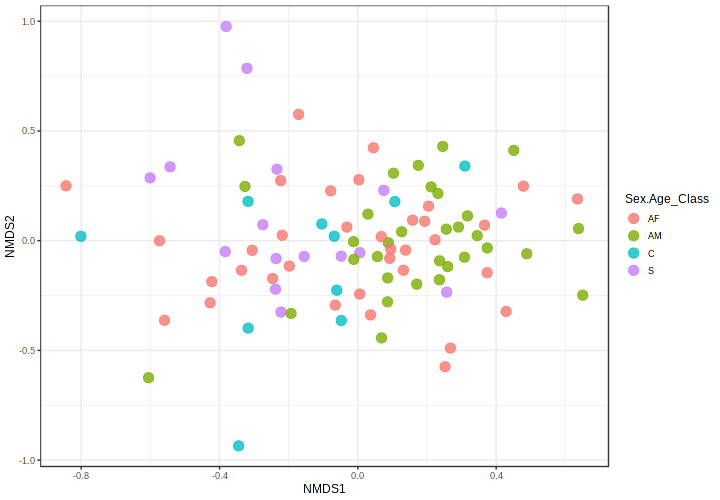


1.0

0.5

0.0

-0.5

-1.0

-0.8

-0.4

0.0

0.4

NMDS 2

**Stress = 0.222**

**Stress = 0.226**


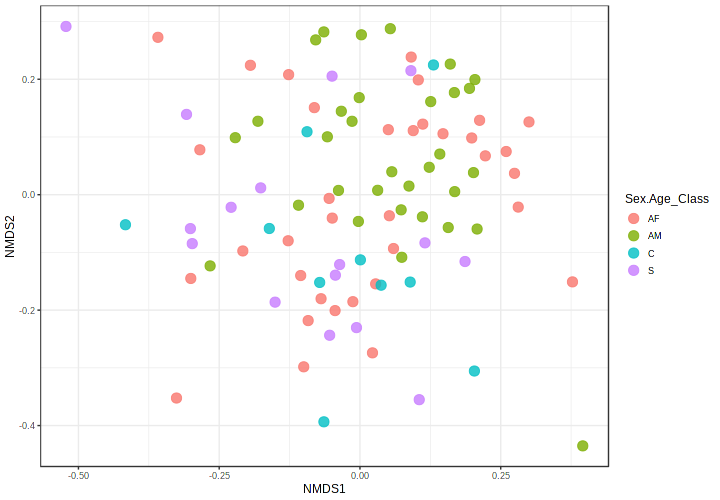


-0.5

-0.25

0.00

0.25

0.2

0.0

-0.2

-0.4

NMDS 1

NMDS 2

**Stress = 0.261**


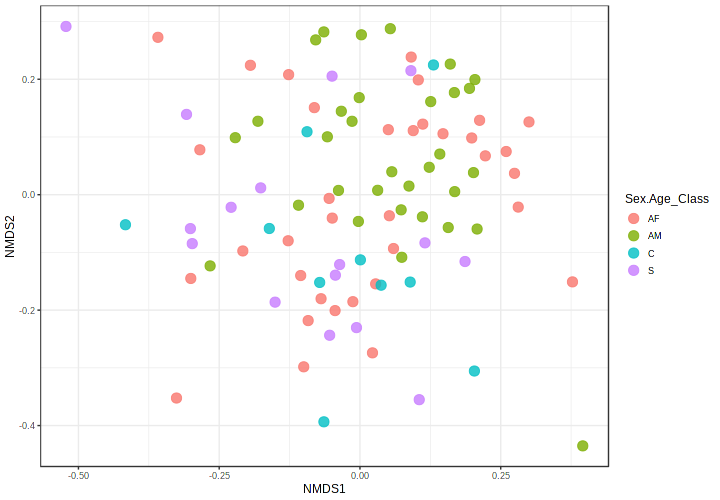


B)

A)

C)


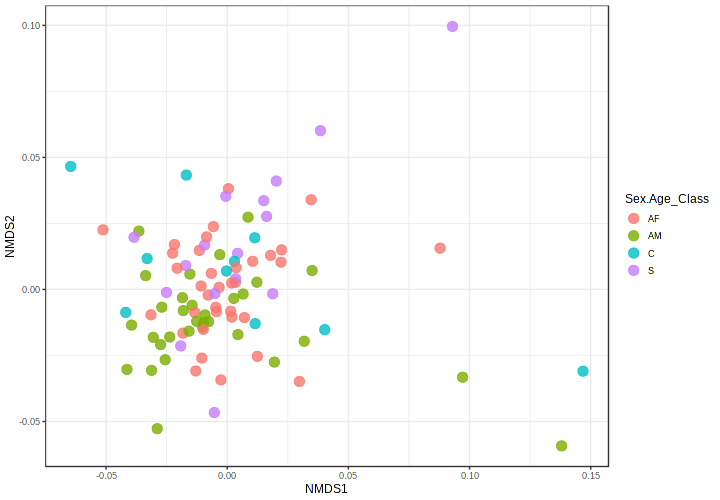


-0.05

-0.0

0.05

0.10

0.15

0.10

0.05

0.00

-0.05

NMDS 1

D)

**Stress = 0.261**


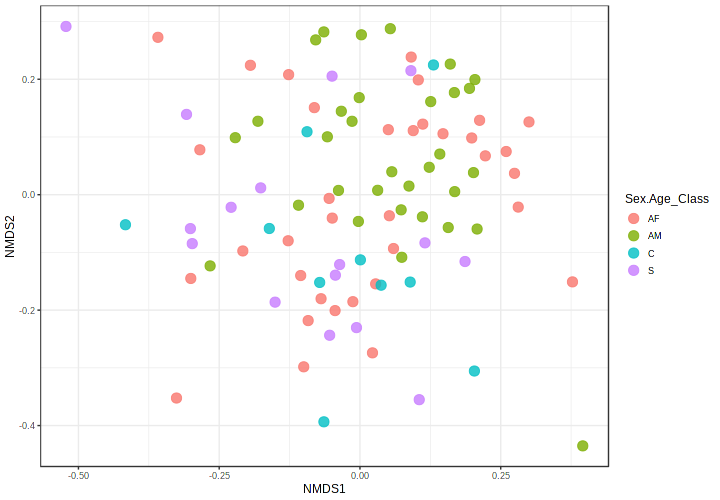

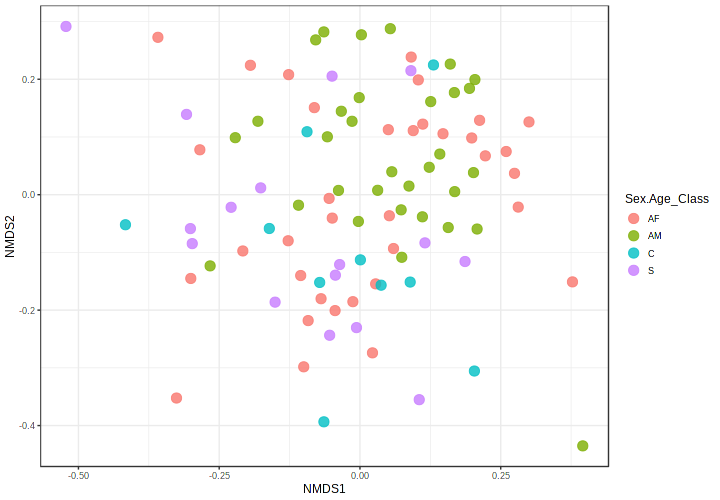

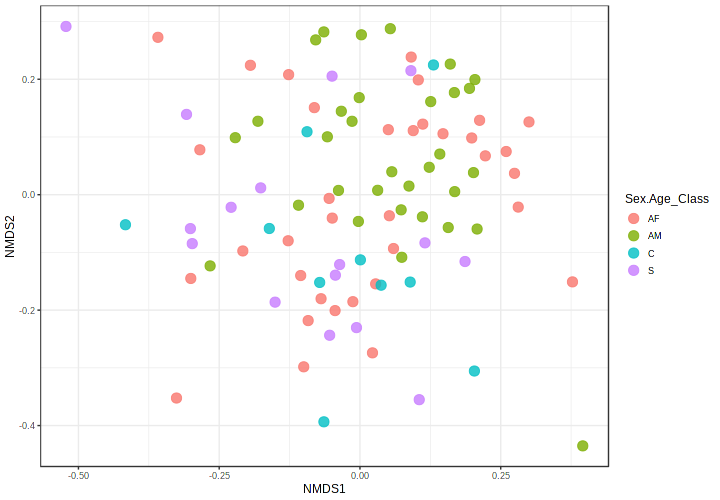

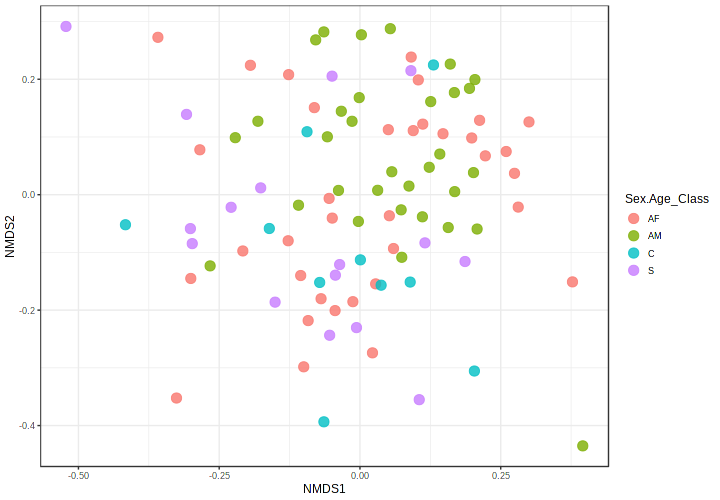


**Supplementary Figure S3.** Non-metric multi-dimensional scaling (NMDS) plots showing gut bacterial community composition differences among sex/age classes (Adult females [AF], adult males [AM], subadults [S], and cubs [C]) for East Greenland (EG) and Southern Beaufort Sea (SB) polar bears using Bray-Curtis distances at bacterial (A) class, (B) genus, and (C) amplicon sequence variant (ASV)-levels and using weighted UniFrac distance at (D) ASV-level.

**Supplementary Figure S4**. Boxplots showing differential abundances of log-transformed class ounts indicating (A) higher Parcubacteria in adult females [AF] (Mean: 0.35 ± 0.2) , adult males [AM] (Mean: 0.5 ± 0.2) and subadults [S] (Mean: 0.07 ± 0.07) compared to cubs [C] (Not detected) and (B) higher Saccharimonadia in AF (Mean: 4.7 ± 0.6) and C (Mean: 5.0 ± 1.0) compared to AM (Mean: 1.5 ± 0.5) and S (3.2 ± 0.7), and (C) higher Bacilli in AF (Mean: 12.2 ± 0.4) and C (Mean: 13.4 ± 0.5) compared to AM (Mean: 10.0 ± 2.7) and S (Mean: 11.1 ± 0.4) sex/age classes for East Greenland (EG) and Southern Beaufort Sea (SB) polar bears. Analysis of composition with bias correction (ANCOM-BC) test results summarized in Supplementary Table S5.

A)

B)

C)


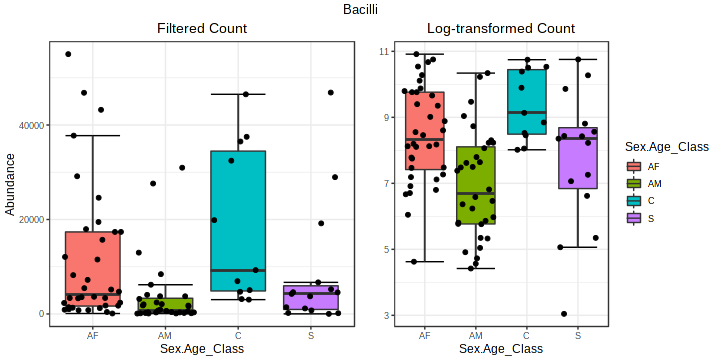

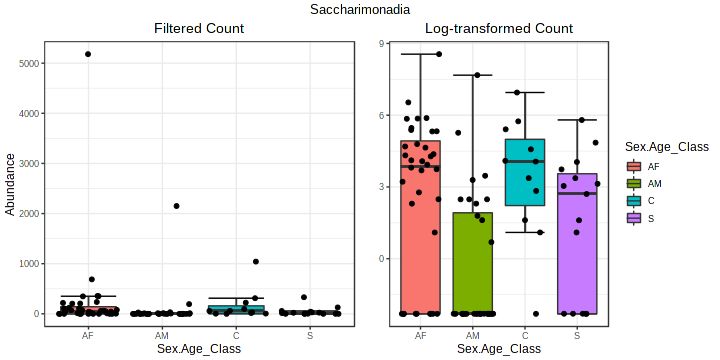

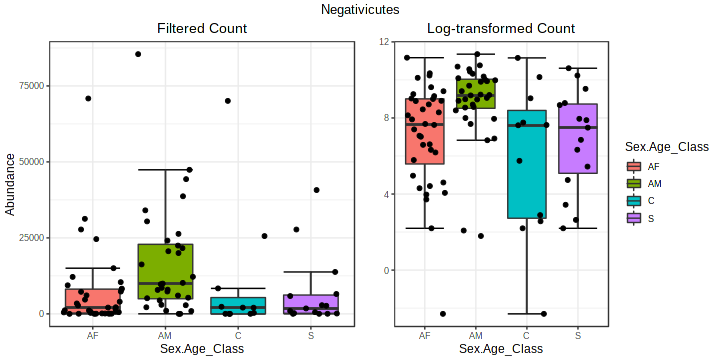


4

2

0

9

6

3

0

11

9

7

5

3

AF

AM

C

S

AF

AM

C

S

AF

AM

C

S

| Fatty Acid | PC1 loading | PC2 loading |
| --- | --- | --- |
| c16.3w6 | 0.373 | -0.194 |
| c18.2w6 | 0.376 | 0.0182 |
| c18.3w3 | 0.394 | -0.133 |
| c20.1w9 | 0.311 | 0.428 |
| c20.5w3 | 0.279 | -0.352 |
| c22.1w11 | 0.143 | 0.567 |
| c22.1w9 | 0.265 | 0.499 |
| c21.5w3 | 0.387 | -0.24 |
| c22.6w3 | 0.386 | -0.097 |

**Supplementary Figure S5. (**A) Principal components analysis (PCA) showing loadings of the top nine fatty acids (FAs) which explained approximately 83.3% of the variation in diet among the 46 Southern Beaufort Sea (SB) samples for which diet data was available. (B) Table listing the top nine FAs and their associated PC1 and PC2 loading scores.


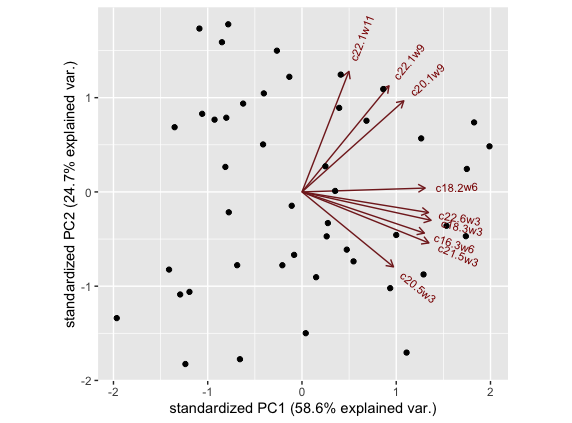


B)

A)

A)

B)

D)

E)

C)

BC NMDS2

Shannon

FPD

Inv. Sim.

BC NMDS1


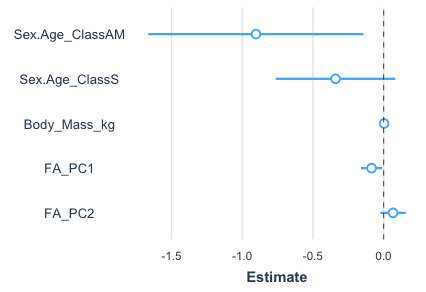

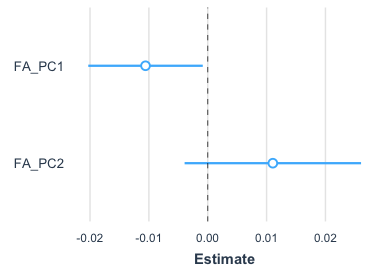

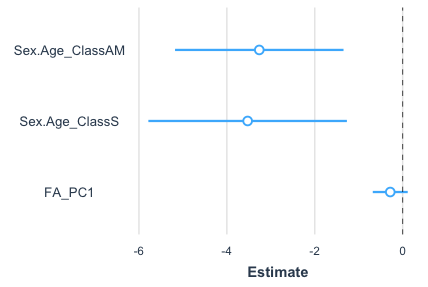

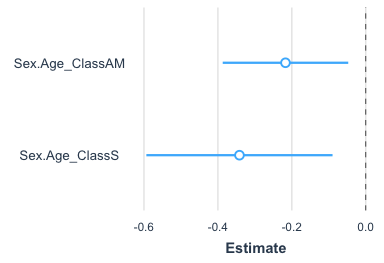

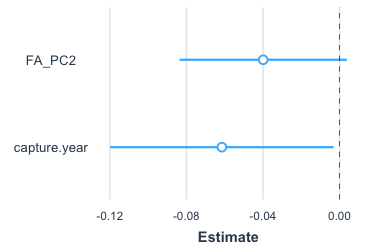


**Supplementary Figure S6.**  Horizontal bar plots showing coefficient values and 95% confidence intervals for linear regression models incorporating diet as a driver of alpha and beta diversity within the subset of Southern Beaufort Sea (SB) polar bears for which diet data was available. (A) Shannon alpha diversity, (B) Inverse Simpson alpha diversity, (C) Faith’s phylogenetic diversity (FPD), (D) first Non-metric multidimensional scaling (NMDS) axis for Bray-Curtis distance (BC NDMDS1), second NMDS axis for Bray-Curtis distance (BC NMDS2). There were no significant terms in models explaining variation in weighted UniFrac NMDS coordinates for the subset of SB polar bears.

**Supplementary Text S1**. *Influence of sex/age class and body mass on gut bacterial composition and diversity for SB subset of polar bears*

In addition to diet, other ecological drivers of variation in gut bacterial composition and diversity were identified. Sex/age class was identified as a significant term in models explaining variation in alpha diversity for Shannon and Faiths phylogenetic diversity (FPD) indices (Table 2, Supplementary Table S8). Adult males and subadult bears appear to have lower Shannon Diversity and FPD compared to adult females (Supplementary Fig. S6A, S6C). Results of the PERMANOVAs found significant gut bacterial compositional differences among SB polar bear sex/age classes at the bacterial genus level and near-significant differences among sex/age classes at the ASV-level (Table 3). A total of 25 bacterial genera and 99 ASVs were differentially abundant among the different sex/age classes (Supplementary Tables S9 and S10). Adult males and subadult bears also appear to load lower on the Bray-Curtis NMDS1 axis compared to adult female bears (Supplementary Fig. S6D).
